# Synthesis, Anticancer Activity, and Molecular Docking of New 1,2,3-Triazole Linked Tetrahydrocurcumin Derivatives

**DOI:** 10.3390/molecules29133010

**Published:** 2024-06-25

**Authors:** Meitao Duan, Ahmed Mahal, Anas Alkouri, Chen Wang, Zhiqiang Zhang, Jungang Ren, Ahmad J. Obaidullah

**Affiliations:** 1 School of Pharmacy, Xiamen Medical College, Xiamen 361023, China; dmt@xmmc.edu.cn (M.D.); wc@xmmc.edu.cn (C.W.); zzq@xmmc.edu.cn (Z.Z.); jqr9422@126.com (J.R.); 2 Xiamen Medical College Research Center for Sustained and Controlled Release Formulations, Xiamen Medical College, Xiamen 361023, China; 3 Key Laboratory of Functional and Clinical Translational Medicine, Fujian Province University, Xiamen Medical College, Xiamen 361023, China; 4 Department of Medical Biochemical Analysis, College of Health Technology, Cihan University-Erbil, Erbil 44001, Kurdisan Region, Iraq; 5 College of Pharmacy, Cihan University-Erbil, Erbil 44001, Kurdisan Region, Iraq; anas.alkhouri@cihanuniversity.edu.iq; 6 Department of Pharmaceutical Chemistry, College of Pharmacy, King Saud University, P.O. Box 2457, Riyadh 11451, Saudi Arabia; aobaidullah@ksu.edu.sa

**Keywords:** tetrahydrocurcumin, click reaction, triazole, anticancer, drug discovery

## Abstract

Cancer is one of the deadliest diseases to humanity. There is significant progress in treating this disease, but developing some drugs that can fight this disease remains a challenge in the field of medical research. Thirteen new 1,2,3-triazole linked tetrahydrocurcumin derivatives were synthesized by click reaction, including a 1,3-dipolar cycloaddition reaction of tetrahydrocurcumin baring mono-alkyne with azides in good yields, and their in vitro anticancer activity against four cancer cell lines, including human cervical carcinoma (HeLa), human lung adenocarcinoma (A549), human hepatoma carcinoma (HepG2), and human colon carcinoma (HCT-116) were investigated using MTT(3-(4,5-dimethylthiazole-2-yl)-2,5-diphenyltetraz-olium bromide) assay. The newly synthesized compounds had their structures identified using NMR HRMS and IR techniques. Some of prepared compounds, including compounds **4g** and **4k**, showed potent cytotoxic activity against four cancer cell lines compared to the positive control of cisplatin and tetrahydrocurcumin. Compound **4g** exhibited anticancer activity with a IC_50_ value of 1.09 ± 0.17 μM against human colon carcinoma HCT-116 and 45.16 ± 0.92 μM against A549 cell lines compared to the positive controls of tetrahydrocurcumin and cisplatin. Moreover, further biological examination in HCT-116 cells showed that compound **4g** can arrest the cell cycle at the G1 phase. A docking study revealed that the potential mechanism by which **4g** exerts its anti-colon cancer effect may be through inhabiting the binding of APC–Asef. Compound **4g** can be used as a promising lead for further exploration of potential anticancer agents.

## 1. Introduction

One of the deadliest diseases worldwide is cancer, a disease in which the cells grow out of control. It causes economic and life losses. Therefore, there is an urgent need for the development of new potential anticancer agents [1]. Modification of natural products is considered one of potential methods leading to potent anticancer agents with good properties, including low resistance and toxicity [2,3]. Small molecules which are produced by any organism involving primary and secondary metabolites are called natural products, and these molecules have been widely used in the development of medicinal chemistry and drug discovery [4,5]. Curcumin is one of the important polyphenol compounds that exist in in the rhizome of Curcuma longa [6]. Curcumin has many potent bioactivities and acts as an antioxidant [7,8], anti-inflammatory [9], neuroprotective [10,11,12], antibacterial [13,14], and anticancer agent [8,15]. Curcumin has a unique symmetrical structure in which O-methoxyphenols are attached to a β-diketone moiety [16,17]. Hydrolysis and oxidation can cause in vitro and in vivo degradation of curcumin [18,19,20]. Tetrahydrocurcumin is one of the compounds that was found as a result of the transformation of curcumin at a physiological “pH” [11,21]. Tetrahydrocurcumin is a traditional Chinese medicine isolated from *Curcuma wenyujin* [22] which is found in turmeric, and tetrahydrocurcumin can be produced from the reduction of curcumin; in the laboratory, it can be prepared from the hydrogenation of curcumin [23]. Tetrahydrocurcumin has displayed potent anticancer activity against human breast cancer [24], cervical cancer [25], Leukemia [26], lung cancer [27], tumor metastasis [28], and colon carcinogenesis [29]. In physiological conditions and due to the limit of the α,β-double bond conjugated to the carbonyl group, tetrahydrocurcumin is more stable than curcumin, which has been found to not have efficient bioactivity because of its instability, high reactivity, and low bioavailability [30,31]. A few reports have demonstrated the synthesis and bioactivity of tetrahydrocurcumin [32,33,34,35,36], such as the preparation of 1,4-dihydropyridine tetrahydrocurcumin derivatives [11]. Nitrogen-containing heterocycles are considered to have strong bioactivity and are very important in the field of medicinal chemistry and drug discovery [37,38,39,40]. A five-membered ring with three nitrogen and two carbon atoms called triazole has demonstrated anticancer activity [41]. The 1,3-dipolar cycloaddition reaction between alkyne and azide lead to the formation of a triazole moiety [42], a promising scaffold for the development of various potent anticancer agents [43,44,45]. In the present work, as part of our search for novel potential bioactive agents [46,47,48,49,50], we report here on the synthesis of novel tetrahydrocurcumin derivatives that bear triazole moiety and are evaluated for their anticancer activities against four human cancer cell lines in vitro.

## 2. Results and Discussion

**Scheme 1:**
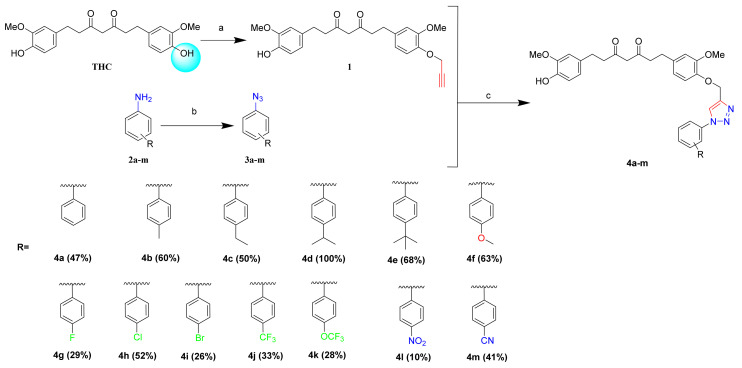
Synthesis, structure, and numbering (as used in NMR signal assignment) of THC-triazole conjugates (**4a–m**). Reagents and conditions: (a) K_2_CO_3_, DMF, propargyl bromide, N_2_, r.t. 24 h; (b) ice bath, sodium azide, trichloromethane, trifluoroacetic acid, r.t. overnight; (c) THF, t-BuOH-H_2_O, copper (II) sulfate pentahydrate, sodium ascorbate, r.t. 12 h.

Scheme 1 summarizes the general procedure for the synthesis of tetrahydrocurcumin derivatives. Tetrahydrocurcumin was alkylated with propargyl bromide in the presence of potassium carbonate (K_2_CO_3_) using dimethylformamide (DMF) at room temperature in an inert atmosphere, resulting in compound **1**. Various azides (**3a**–**m**) were obtained by the reaction of anilines (**2a**–**m**) with sodium azide using dichloromethane (DCM) as a solvent [51]. Click reaction was utilized for the synthesis of the tetrahydrocurcumin conjugates (**4a**–**m**) by the reaction between compound 1 and azides using copper (II) sulfate pentahydrate (CuSO_4_•5H_2_O), monosodium ascorbate in THF (2 mL), and a mixture of *t*-BuOH:H_2_O (2 mL, *v:v* = 1:1) at room temperature for 12 h. The column chromatography technique was used for purification of the newly prepared compounds (**4a**–**m**) by utilizing a mixture of DCM/EtOAc and ^1^H-NMR. ^13^C-NMR, HR-ESI-MS, and IR techniques were used for identification of their structures. ^1^The H-NMR spectrum of compounds indicated four singlets signals ranging from 7.60 to 7. 7.22 for the new aryl ring. ^1T^he H-NMR spectrum of the compounds indicated two singlets signals ranging from 3.85 to 3.76 ppm corresponding to two OCH_3_ and three singlet signals corresponding to three OCH_3_ groups (compound **4f**). ^1^The H-NMR spectrum of compounds indicated one singlet signal ranging from 8.20 to 8.00 ppm corresponding to one CH of the triazole group. ^13^The C-NMR of the compounds revealed the presence of signals ranging from 56.20 to 55.60 ppm corresponding to three OCH_3_ groups. ^13^The C-NMR of the compounds revealed the presence of signals ranging from 144.2 to 120.0 in the triazole ring. FTIR showed signals for OH (3300–3380 cm^−1^), CO (1600–1670 cm^−1)^) and C=N (1500–1570 cm^−1^). 

The compounds were evaluated for their anticancer activity against four cancer cell lines including human cervical carcinoma (HeLa), human lung adenocarcinoma (A549), human hepatoma carcinoma (HepG2), and human colon carcinoma (HCT-116) using an MTT(3-(4,5-dimethylthiazole-2-yl)-2,5-diphenyltetraz-olium bromide) assay. Cisplatin and tetrahydrocurcumin were used as positive controls. A concentration of 50% was used for cell growth inhibition (IC_50_), which measured the anticancer activity of the compounds (**4a**–**m**). Table 1 summarizes the anti-proliferative activity data of the prepared compounds (**4a**–**m**) by IC_50_ values. 

The compounds **4b**–**4d** and **4l** showed no significant cytotoxic activity against all four cancer cell lines compared to the positive controls of tetrahydrocurcumin and cisplatin. Compounds **4a**, **4h**, **4j**, and **4k** exhibited moderate anticancer activity against the HCT116 cell line, with IC_50_ values in ranging from 62.63 to 89.38 μM compared to tetrahydrocurcumin while showing no anticancer activity compared to cisplatin. Compounds **4g**, **4j**, and **4k** displayed lower anti-proliferative activities against the HeLa cell line, with IC_50_ values ranging from 129.50 to 190.00 μM compared to both tetrahydrocurcumin and cisplatin. Compounds **4e**, **4j**, and **4m** showed no potent anticancer activity against the A549 cell line, with IC_50_ values ranging from 131.20 to 162.40 μM compared to both positive controls of tetrahydrocurcumin and cisplatin. Compound **4k** showed weak anticancer activity against the HepG2 cell line, with an IC_50_ value of 104.23 μM compared to the positive controls of tetrahydrocurcumin and cisplatin. Compound **4f** showed potent cytotoxic activity against HCT116 and HepG2 cancer cell lines, with IC_50_ values of 15.59 and 53.64 μM compared to tetrahydrocurcumin and cisplatin. Compound **4k** displayed significant anticancer activity (against the A549 cancer cell line compared to tetrahydrocurcumin, while showing moderate anticancer activity (IC_50_ = 57.96 μM). Compound **4g** showed strong cytotoxic activity against the HCT117 and HepG2 cancer cell lines, with IC_50_ values of 1.09 and 66.82 μM, respectively, compared to tetrahydrocurcumin and cisplatin, while showing moderate anticancer activity compared to A549 compared to tetrahydrocurcumin and cisplatin. The data showed that the incorporation of an electron-withdrawing group into the benzene ring led to significant improvement in cytotoxic activity compared to the electron-donating group. Through the prediction of the properties of the target compounds (Table 2), it was found that the molecular weight of the derivatives is greater than 500 (529.59~613.59), the lipid water distribution coefficient is greater than 3 (3.33~5.28), the total number of hydrogen bond acceptors and donors is greater than 10 (10–11), the number of rotatable bonds is 14–15, the molecular polarity range is 112.79~136.58, and the molecular volume is greater than 400 (482.19~613.59). The properties of derivatives do not comply with Lipinski’s “rule of five” but cater to the properties of small-molecule inhibitors of protein–protein interactions (PPIs).

**Figure 1:**
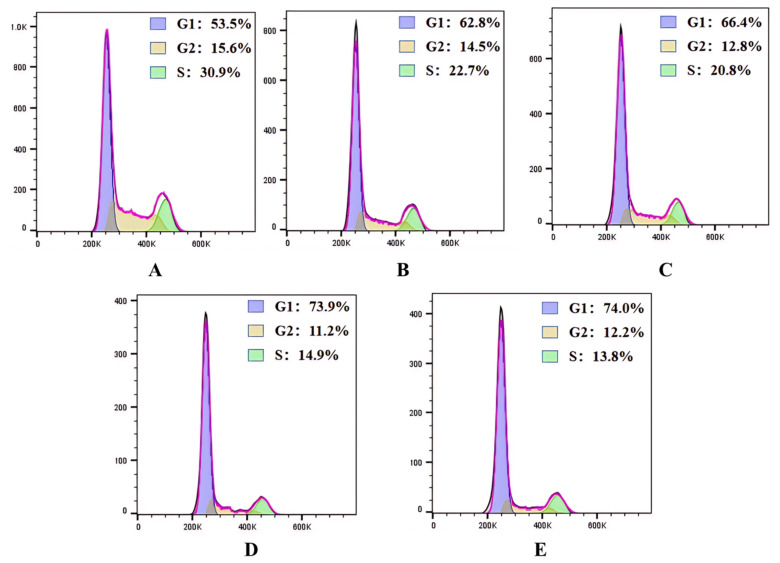
Compound **4g** arrests the cell cycle in HCT-116 cells. Flow cytometry analysis of HCT-116 cells stained with propidium iodide and treated with **4g** for 72 h. (**A**) Control group; (**B**) Compound **4g**, 1.56 μM; (**C**) Compound **4g**, 3.125 μM; (**D**) Compound **4g**, 6.25 μM; (**E**) Compound **4g**, 12.5 μM; Compound **4g**, 25 μM.

The HCT-116 cell lines were treated with compounds **4g** in order to evaluate the role of cell cycle arrest of GA derivatives, which caused growth inhibition. The cell cycle distribution was evaluated by flow cytometric analysis after staining of cellular DNA with propidium iodide at the different concentrations of compound **4g** (1.56 μM~25 μM). As shown in Figure 1, the percentage of cells in the G1 phase of the cell cycle increased from 53.5% to 74.0%, the S phase decreased from 30.9% to 13.8%, and the G2 phase decreased from 15.6% to 12.2% after 72 h of treatment. The obtained result indicated that compound **4g** induced accumulations in the G phase (DNA synthesis pre stage) of the cell cycle. 

**Figure 2:**
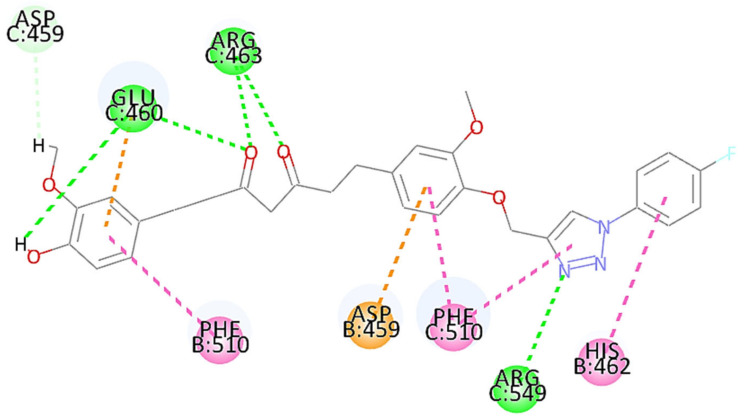
Interaction between **4g** and APC–Asef crystal protein.

The binding of APC and Asef plays an important role in the development of colon cancer, and their binding belongs to the protein–protein interaction process. Considering the significant effect of compound **4g** against HCT116 and its compliance with the properties of small-molecule PPIs, we further evaluated the interaction between compound **4g** and APC–Asef using molecular docking techniques. The docking results showed that there are interactions between **4g** and the APC–Asef crystal protein, mainly through hydrogen bonding and hydrophobic interactions (Figure 2). Among them, the aspartic acid (ASP^459^), glutamic acid (GLU^460^), arginine (ARG^463^), and arginine (ARG^549^) of the APC–Asef crystal protein interact with the C7 methoxy group (OCH_3_), C5 hydroxyl group (OH)/C10 carbonyl group (C=O), C10 ′carbonyl group (C=O), and triazole of **4g** to form hydrogen bonds, respectively; The Aspartate (ASP^459^), histidine (HIS^462^), and phenylalanine (PHE^510^) form hydrophobic interactions with the benzene and triazole rings of **4g**. Based on the results, it is speculated that the potential mechanism by which **4g** exerts its anti-colon cancer effect may be through inhibiting the binding of APC–Asef.

## 3. Experimental Procedure

### 3.1. General Experimental Procedures

Commercial sources of reagents and solvents were purchased from Shanghai Bide Pharmaceutical Technology Co., Ltd. (Shanghai, China), Shanghai Macklin Biochemical Co., Ltd. (Shanghai, China), Guangzhou Chemical Reagent (Guangzhou, China), and Tianjin Damao Chemical Reagent Factory (Guangzhou, China). Shanghai D&B Biological Science and Technology Co., Ltd. (Shanghai, China). and Yantai Jiangyou Silica Gel Development Co., Ltd. (Yantai, China) were the sources of tetrahydrocurcumin and silica gel (200~300 mesh), respectively. UPLC data were collected using an Agilent 1290 Infinity II HDR-DAD instrument (Agilent, Santa Clara, CA, USA) and HRMS data were obtained by utilizing the ESI mode on an Orbitrap Eclipse device (Thermo Fisher Scientific, Waltham, MA, USA). An AVANCE IIITM HD 600 MHz device was used for the measurement of the NMR data, where the coupling constants (*J*) are in Hz and the chemical shifts (δ) are given in ppm. The abbreviations used for the splitting pattern are as follows: s = singlet, d = doublet, dd = doublet of doublets, ddd = doublet of doublet of doublets, t = triplet, m = multiplet, and brs = broad singlet. 

#### 3.1.1. General Procedure for the Synthesis of 1-(4-Hydroxy-3-methoxyphenyl)-7-(3-methoxy-4-(prop-2-yn-1-yloxy)phenyl)heptane-3,5-dione (**1**)

Potassium carbonate (1.1 eq) and propargyl bromide were added to the THC (1.0 eq) dissolved in DMF (5 mL), and the reaction was allowed to stir at room temperature overnight. After the reaction, add 15 mL of water, extract with dichloromethane, dry with anhydrous sodium sulfate, filter, evaporate under reduced pressure to remove solvent, and purify with silica gel column chromatography to give product **1**.

#### 3.1.2. General Procedure for the Synthesis of Azide Derivatives (**3a**~**m**)

Aniline (**2a**~**m**) (1.0 eq) was dissolved in CH_3_CN (5 mL) in a 25 mL round-bottomed flask and cooled to 0 °C in an ice bath. To this stirred mixture was added *t*-BuONO (1.5 eq) followed by TMSN_3_ (1.2 eq) dropwise. The resulting solution was stirred at room temperature for 1 h. The reaction mixture was concentrated under vacuum and extracted with DCM (10 mL × 3) to give product **3a**~**m** [50,51].

#### 3.1.3. General Procedure for the Synthesis of 1,2,3-Riazole Linked THC Derivatives (**4a**~**m**)

To a solution of product **1** (1.2 eq) add aromatic azides (**3a**~**m**) (1.0 eq) in THF (2 mL) and *t*-BuOH-H_2_O (2 mL, *v*:*v* = 1:1) at room temperature and then add copper (II) sulfate pentahydrate (20 mg) and sodium ascorbate (2 g in 10 mL H_2_O, 0.6 mL). The reaction mixture must stir at room temperature till completion of the reaction (monitored by TLC). The solvent is evaporated and residue is chromatographed on silica gel using Petroleum Ether:EtOAC to offer the target **4a**~**m** [50,51].

#### 3.1.4. 1-(4-Hydroxy-3-methoxyphenyl)-7-(3-methoxy-4-((1-phenyl-1H-1,2,3-triazol-4-yl)methoxy) phenyl)heptane-3,5-dione (**4a**)

Yellow oil, (139.52 mg, 0.340 mmol, 47%). IR (cm^−1^): 3313 (OH), 1600 (C=O), 1540 (C=N); ^1^H-NMR (600 MHz, CDCl_3_) δ 8.07 (s, 1H, triazole-H), 7.65–7.62 (dd, *J* = 7.8, 15.6 Hz, 3H, Ar-H), 7.52–7.48 (q, *J* = 7.8, 16.8 Hz, 2H, Ar-H), 6.79–6.76 (m, 2H, Ar-H), 6.66–6.56 (m, 4H, Ar-H), 5.48 (s, 2H, Et-H), 3.83 (s, 6H, OMe-H), 2.87–2.72 (m, 10H, Et-H). ^13^C-NMR (150 MHz, CDCl_3_) δ 206.21, 204.62, 146.53, 144.10, 132.92, 132.48, 129.89, 129.83, 128.87, 128.80, 125.01, 121.24, 121.10, 120.95, 120.51, 120.48, 119.15, 114.44, 114.39, 111.50, 111.21, 111.15, 70.28, 66.24, 56.06, 56.01, 44.70, 42.04, 29.16, 29.08. ESI-HRMS Calcd for C_30_H_32_N_3_O_6_ [M + H]^+^: 530.2286, found 530.2291.

#### 3.1.5. 1-(4-Hydroxy-3-methoxyphenyl)-7-(3-methoxy-4-((1-(p-tolyl)-1H-1,2,3-triazol-5-yl)methoxy) phenyl)heptane-3,5-dione (**4b**)

Yellow oil, (235.10 mg, 0.436 mmol, 60%). IR (cm^−1^): 3352 (OH), 1590 (C=O), 1522 (C=N); ^1^H-NMR (400 MHz, CDCl_3_) δ 8.04 (s, 1H, triazole-H), 7.53 (d, 2H, *J *= 14.4 Hz, Ar-H), 7.31 (d, 2H, *J *= 14.0 Hz, Ar-H), 6.71–6.79 (m, 3H, Ar-H), 6.67–6.59 (m, 3H, Ar-H), 5.60 (s, 2H, Et-H), 3.86 (s, 6H, OMe-H), 2.98~2.43 (m, 10H, Et-H), 2.44 (s, 3H, Me-H). ^13^C-NMR (100 MHz, CDCl_3_) δ 206.30, 204.66, 162.71, 146.56, 144.79, 144.10, 143.06, 138.96, 138.86, 134.69, 132.91, 132.48, 130.34, 130.28, 121.46, 121.20, 120.93, 120.38, 120.24, 114.45, 111.50, 111.17, 70.30, 66.29, 56.05, 56.00, 44.69, 42.04, 29.14, 29.08, 21.20. ESI-HRMS Calcd for C_31_H_34_N_3_O_6_ [M + H]^+^: 544.2442, found 544.2439. 

#### 3.1.6. 1-(4-((1-(4-Ethylphenyl)-1H-1,2,3-triazol-4-yl)methoxy)-3-methoxyphenyl)-7-(4-hydroxy-3-methoxyphenyl)heptane-3,5-dione (**4c**)

Yellow oil, (203.75 mg, 0.368 mmol, 50%). IR (cm^−1^): 3324 (OH), 1521 (C=O), 1509 (C=N); ^1^H-NMR (500 MHz, CDCl_3_) δ 8.00 (s, 1H, triazole-H), 7.55–7.51 (dd, *J *= 8.5, 10.5 Hz, 2H, Ar-H), 7.29–7.32 (t, *J* = 8.0 Hz, 2H, Ar-H), 6.78–6.76 (t, *J* = 7.0 Hz, 2H, Ar-H), 6.64–6.57 (m, 4H, Ar-H), 5.58 (d, *J* = 10.5 Hz, 2H, Et-H), 3.82 (d, *J* = 2.0 Hz, 6H, OMe-H), 2.82–2.70 (m, 12H, Et-H), 1.26 (td, *J* = 7.5, 1.5 Hz, 3H, Me-H). ^13^C-NMR (126 MHz, CDCl_3_) δ 206.30, 204.66, 146.52, 145.26, 145.17, 144.76, 144.06, 143.03, 134.79, 132.88, 132.45, 129.16, 129.10, 121.47, 121.17, 120.90, 120.47, 120.45, 120.24, 114.42, 111.45, 111.13, 70.27, 66.25, 56.00, 55.96, 44.67, 42.01, 29.10, 29.05, 28.54, 15.55. ESI-HRMS Calcd for C_32_H_36_N_3_O_6_ [M + H]^+^: 558.2599, found 558.2604.

#### 3.1.7. 1-(4-Hydroxy-3-methoxyphenyl)-7-(4-((1-(4-isopropylphenyl)-1H-1,2,3-triazol-4-yl)methoxy)-3-methoxyphenyl)heptane-3,5-dione (**4d**)

Yellow oil, (429.83 mg, 0.746 mmol, 100%). IR (cm^−1^): 3331 (OH), 1504 (C=O), 1539 (C=N); ^1^H-NMR (500 MHz, CDCl_3_) δ 8.00 (s, 1H, triazole-H), 7.54–7.52 (dd, *J *= 8.5, 10.5 Hz, 2H, Ar-H), 7.32–7.30 (t, *J* = 8.0 Hz, 2H, Ar-H), 6.78–6.76 (d, *J* = 7.0 Hz, 2H, Ar-H), 6.64–6.57 (m, 4H, Ar-H), 5.58 (d, *J* = 10.5 Hz, 2H, Et-H), 3.81 (d, *J* = 2.0 Hz, 6H, OMe-H), 2.87 (s, 1H, CH-H), 2.82–2.68 (m, 10H, Et-H),1.26 (m, 6H, Me-H). ^13^C-NMR (126 MHz, CDCl_3_) δ 206.30, 204.66, 146.52, 145.26, 145.17, 144.76, 144.06, 143.03, 134.79, 132.88, 132.45, 129.16, 129.10, 121.47, 121.17, 120.90, 120.47, 120.45, 120.24, 114.42, 111.45, 111.13, 70.27, 66.25, 56.00, 55.96, 44.67, 42.01, 29.10, 29.05, 28.54, 27.00, 23.93. ESI-HRMS Calcd for C_33_H_38_N_3_O_6_ [M + H]^+^: 572.2755, found 572.2740. 

#### 3.1.8. 1-(4-((1-(4-(Tert-butyl)phenyl)-1H-1,2,3-triazol-4-yl)methoxy)-3-methoxyphenyl)-7-(4-hydroxy-3-methoxyphenyl)heptane-3,5-dione (**4e**)

Yellow oil, (289.39 mg, 0.498 mmol, 68%). IR (cm^−1^): 3345 (OH), 1542 (C=O), 1527 (C=N); ^1^H-NMR (400 MHz, CDCl_3_) δ 8.03 (s, 1H, triazole-H), 7.56–7.53 (d, *J* = 12.0 Hz, 4H, Ar-H), 6.80–6.65 (m, 6H, Ar-H), 5.64 (s, 2H, Et-H), 3.84 (s, 6H, OMe-H), 2.97–2.77 (m, 10H, Et-H), 1.38 (s, 9H, Me-H). ^13^C-NMR (100 MHz, CDCl_3_) δ 206.24, 204.57, 162.62, 152.01, 146.47, 144.00, 142.96, 134.44, 132.79, 132.36, 126.70, 126.61, 126.55, 121.42, 121.08, 120.82, 120.09, 120.04, 118.63, 114.37, 111.40, 111.08, 70.21, 66.19, 55.93, 55.89, 44.61, 41.95, 34.77, 31.47, 31.34, 31.27, 29.03, 28.99. ESI-HRMS Calcd for C_34_H_40_N_3_O_6_ [M + H]^+^: 586.2912, found 582.2916.

#### 3.1.9. 1-(4-Hydroxy-3-methoxyphenyl)-7-(3-methoxy-4-((1-(4-methoxyphenyl)-1H-1,2,3-triazol-4-yl)methoxy)phenyl)heptane-3,5-dione (**4f**)

Yellow oil, (256.67 mg, 0.462 mmol, 63%). IR (cm^−1^): 3375 (OH), 1518 (C=O), 1528 (C=N); ^1^H-NMR (400 MHz, CDCl_3_) δ 8.02 (s, 1H, triazole-H), 7.57–7.54 (d, *J* = 12.0 Hz, 2H, Ar-H), 7.00–6.63 (m, 8H, Ar-H), 5.84 (s, 2H, Et-H), 3.80 (d, *J* = 13.2 Hz, 9H, OMe-H), 2.96–2.76 (m, 10H, Et-H). ^13^C-NMR (100 MHz, CDCl_3_) δ 206.30, 204.67, 162.70, 159.84, 159.78, 146.60, 144.11, 132.39, 130.42, 122.02, 121.14, 120.99, 120.86, 120.39, 120.05, 115.20, 114.82, 114.77, 114.48, 111.55, 111.27, 111.21, 70.23, 66.22, 55.94, 55.70, 44.62, 36.58, 31.52, 29.07, 23.90. ESI-HRMS Calcd for C_31_H_34_N_3_O_7_ [M + H]^+^: 560.2391, found 560.2381.

#### 3.1.10. 1-(4-((1-(4-Fluorophenyl)-1H-1,2,3-triazol-4-yl)methoxy)-3-methoxyphenyl)-7-(4-hydroxy-3-methoxyphenyl)heptane-3,5-dione (**4g**)

Yellow oil, (114.29 mg, 0.210 mmol, 29%). IR (cm^−1^): 3358 (OH), 1503 (C=O), 1519 (C=N); ^1^H-NMR (400 MHz, CDCl_3_) δ 8.04 (s, 1H, triazole-H), 7.62–7.60 (d, *J* = 11.2 Hz, 2H, Ar-H), 7.30–7.23 (m, 2H, Ar-H), 6.80–6.62 (m, 6H, Ar-H), 5.58 (s, 2H, Et-H), 3.86 (s, 6H, OMe-H), 2.98–2.78 (m, 10H, Et-H). ^13^C-NMR (101 MHz, CDCl_3_) δ 206.09, 204.62, 161.27 (d, J*_F,C_*= 243.4 Hz, CF), 146.55, 144.11, 132.46, 122.48, 122.40, 121.28, 121.11, 121.02, 120.95, 120.48, 120.43, 116.94, 116.88, 116.71, 116.65, 114.45, 111.59, 111.29, 111.19, 66.24, 60.54, 56.07, 56.01, 44.64, 41.97, 29.14, 29.00. ESI-HRMS Calcd for C_30_H_31_FN_3_O_6_ [M + H]^+^: 548.2191, found 548.2182.

#### 3.1.11. 1-(4-((1-(4-Chlorophenyl)-1H-1,2,3-triazol-4-yl)methoxy)-3-methoxyphenyl)-7-(4-hydroxy-3-methoxyphenyl)heptane-3,5-dione (**4h**)

Yellow oil, (212.84 mg, 0.381 mmol, 52%). IR (cm^−1^): 3317 (OH), 1568 (C=O), 1542 (C=N); ^1^H-NMR (400 MHz, CDCl_3_) δ 8.04 (s, 1H, triazole-H), 7.61–7.51 (m, 2H, Ar-H), 6.81–6.65 (m, 2H, Ar-H), 5.61 (s, 2H, Et-H), 3.86 (s, 6H, OMe-H), 2.98–2.78 (m, 10H, Et-H). ^13^C-NMR (100 MHz, CDCl_3_) δ 206.03, 204.59, 146.55, 145.21, 144.11, 143.36, 135.46, 135.38, 134.59, 134.55, 132.44, 130.03, 129.98, 121.66, 121.45, 121.27, 121.09, 120.94, 120.20, 114.46, 111.58, 111.18, 66.20, 60.54, 56.06, 56.00, 44.63, 41.95, 29.13, 28.98. ESI-HRMS Calcd for C_30_H_31_ClN_3_O_6_ [M + H]^+^: 564.1896, found 564.1894.

#### 3.1.12. 1-(4-((1-(4-Bromophenyl)-1H-1,2,3-triazol-4-yl)methoxy)-3-methoxyphenyl)-7-(4-hydroxy-3-methoxyphenyl)heptane-3,5-dione (**4i**)

Yellow oil, (113.91 mg, 0.189 mmol, 26%). IR (cm^−1^): 3353 (OH), 1544 (C=O), 1531 (C=N); ^1^H-NMR (400 MHz, CDCl_3_) δ 8.04 (s, 1H, triazole-H), 7.65–7.56 (m, 4H, Ar-H), 6.79–6.64 (m, 6H, Ar-H), 5.45 (s, 2H, Et-H), 3.85 (s, 6H, OMe-H), 2.98–2.77 (m, 10H, Et-H). ^13^C-NMR (101 MHz, CDCl_3_) δ 204.58, 162.76 (d, J*_F,C_*= 241.5 Hz, CF), 146.56, 144.12, 135.93, 132.99, 132.94, 132.89, 132.42, 124.21, 122.45, 121.82, 121.25, 120.93, 120.78, 120.17, 114.46, 114.36, 111.20, 111.09, 66.18, 56.07, 56.00, 44.62, 36.64, 31.59, 31.41, 29.12, 28.96, 23.86. ESI-HRMS Calcd for C_30_H_31_BrN_3_O_6_ [M + H]^+^: 608.1391, found 608.1397.

#### 3.1.13. 1-(4-Hydroxy-3-methoxyphenyl)-7-(3-methoxy-4-((1-(4-(trifluoromethyl)phenyl)-1H-1,2,3-triazol-4-yl)methoxy)phenyl)heptane-3,5-dione (**4j**)

Yellow oil, (144.05 mg, 0.243 mmol, 28%). IR (cm^−1^): 3340 (OH), 1504 (C=O), 1515 (C=N); ^1^H-NMR (400 MHz, CDCl_3_) δ 8.00 (s, 1H, triazole-H), 7.68–7.67 (d, *J*=7.2 Hz, 2H, Ar-H), 7.37–7.35 (d, *J* = 7.2 Hz, 2H, Ar-H), 6.76–6.75 (d, *J* = 6.4 Hz, 3H, Ar-H), 6.61 (s, 3H, Ar-H), 5.63 (s, 2H, Et-H), 3.82 (s, 6H, OMe-H), 2.75–2.73 (m, 10H, Et-H). ^13^C-NMR (101 MHz, CDCl_3_) δ 206.73, 197.93, 146.61, 146.49, 144.18, 144.06, 142.07, 138.31, 132.80, 132.41, 132.31, 131.88, 131.79, 129.19, 129.16, 121.22, 120.98, 116.36, 116.14, 114.55, 114.39, 111.41, 111.34, 56.02, 46.01, 40.94, 30.00, 28.94. ESI-HRMS Calcd for C_31_H_31_F_3_N_3_O_6_ [M + H]^+^: 598.2159, found 598.2159.

#### 3.1.14. 1-(4-Hydroxy-3-methoxyphenyl)-7-(3-methoxy-4-((1-(4-(trifluoromethoxy)phenyl)-1H-1,2,3-triazol-4-yl)methoxy)phenyl)heptane-3,5-dione (**4k**)

Yellow oil, (124.49 mg, 0.204 mmol, 33%). IR (cm^−1^): 3376 (OH), 1550 (C=O), 1534 (C=N); ^1^H-NMR (500 MHz, CDCl_3_) δ 8.01 (s, 1H, triazole-H), 7.78–7.66 (m, 4H, Ar-H), 6.76–6.62 (m, 6H, Ar-H), 5.54 (s, 2H, Et-H), 3.83 (s, 6H, OMe-H), 2.95–2.55 (m, 10H, Et-H). ^13^C-NMR (126 MHz, CDCl_3_) δ 204.58, 171.34, 162.76 (d, J*_F,C_* = 2444.1 Hz, CF), 149.01, 146.53, 145.25, 144.08, 135.31, 132.39, 122.37, 121.81, 121.62, 121.47, 121.06, 120.90, 120.64, 120.30, 119.41, 114.44, 114.39, 111.16, 66.15, 60.52, 55.95, 55.89, 44.60, 29.08. ESI-HRMS Calcd for C_31_H_31_F_3_N_3_O_7_ [M + H]^+^: 614.2109, found 614.2100.

#### 3.1.15. 1-(4-Hydroxy-3-methoxyphenyl)-7-(3-methoxy-4-((1-(4-nitrophenyl)-1H-1,2,3-triazol-4-yl)methoxy)phenyl)heptane-3,5-dione (**4l**)

Yellow oil, (40.87 mg, 0.072 mmol, 10%). IR (cm^−1^): 3352 (OH), 1511 (C=O), 1589 (C=N); ^1^H-NMR (400 MHz, CDCl_3_) δ 8.39–8.37 (d, *J* = 7.6 Hz, 2H, Ar-H), 8.00 (s, 1H, triazole-H), 7.86–7.84 (d, *J* = 7.6 Hz, 10.4 Hz, 2H, Ar-H), 6.76–6.57 (m, 6H, Ar-H), 5.53 (s, 2H, Et-H), 3.83 (s, 6H, OMe-H), 2.95–2.70 (m, 10H, Et-H). ^13^C-NMR (101 MHz, CDCl_3_) δ 205.67, 204.49, 147.29, 146.55, 145.88, 144.21, 144.13, 141.09, 140.99, 132.86, 132.39, 125.63, 121.57, 121.35, 121.14, 120.95, 120.54, 120.41, 120.23, 114.46, 111.70, 111.21, 66.09, 60.54, 56.13, 56.02, 44.57, 41.90, 29.14, 28.90. ESI-HRMS Calcd for C_30_H_31_N_4_O_8_ [M + H]^+^: 575.2136, found 575.2149.

#### 3.1.16. 4-(4-((4-(7-(4-Hydroxy-3-methoxyphenyl)-3,5-dioxoheptyl)-2-methoxyphenoxy)methyl)-1H-1,2,3-triazol-1-yl)benzonitrile (**4m**)

Yellow oil, (165.18 mg, 0.300 mmol, 41%). IR (cm^−1^): 3313 (OH), 1596 (C=O), 1552 (C=N); ^1^H-NMR (400 MHz, CDCl_3_) δ 8.00 (s, 1H, triazole-H), 7.80–7.76 (m, 4H, Ar-H), 6.75–6.58 (m, 6H, Ar-H), 5.64 (s, 2H, Et-H), 3.82 (s, 6H, OMe-H), 2.95–2.69 (m, 10H, Et-H). ^13^C-NMR (101 MHz, CDCl_3_) δ 204.41, 162.67, 146.46, 145.62, 144.02, 143.68, 139.62, 133.92, 132.28, 121.28, 121.21, 120.82, 120.47, 120.38, 120.19, 119.96, 117.75, 114.45, 114.37, 112.31, 111.61, 111.12, 65.99, 56.00, 55.91, 44.47, 36.55, 31.49, 29.01, 28.78, 23.71. ESI-HRMS Calcd for C_31_H_31_N_4_O_6_ [M + H]^+^: 555.2238, found 555.2247.

### 3.2. In Vitro Cytotoxicity Activity 

Human lung adenocarcinoma (A549), human cervical carcinoma (HeLa), and human breast carcinoma (MCF-7) cell lines were obtained from Kunming Institute of Zoology, Chinese Academy of Sciences (Kunming, China). The cells were cultured in RPMI 1640 (for A549 and HeLa cells) and DMEM (for MCF-7 cell) medium supplemented with 10% heat-inactivated fetal bovine serum, as well as 1% penicillin and streptomycin in a 37 °C, 5% CO_2_ incubator. Cell viability was estimated by the MTT colorimetric assay, as previously reported [4,5,44]. The test compounds in DMSO (10 mM) were serially diluted with culture medium. Test cells (100 μL, 5 × 10^4^ cells/mL) were seeded into a 96-well microplate and incubated for approximately 24 h; then, the supernatant was removed, and 100 μL of fresh medium and 100 μL of medium containing a test compound or vehicle control (0.5% DMSO) were added. The blank control contained 200 μL of medium without cells. The final concentrations of each compound in the wells were 100, 50, 25, 12.5, 6.25, 3.125, 1.56, and 0.78 μM, and the content of DMSO in each drug treatment was less than 0.5% (*v*/*v*). The experiments for each concentration were performed in quadruplicate. Cisplatin was used as a positive control. Cells were further incubated for 72 h and then treated with MTT (20 μL/well, 5 mg/mL in DMSO). After another 4 h of incubation, the supernatant per well was removed and 150 μL DMSO was added to each well. The optical density (OD) of each well was measured on a Genois microplate reader at a wavelength of 570 nm. The inhibitory rate of cell growth was calculated according to the following formula: Inhibition rate (%) = {1 − [OD (compounds) − OD (blank)]/[OD (controls) − OD (blank)]} × 100%. IC_50_ values were determined by nonlinear regression analysis of logistic dose–response curves (SPSS 17.0 statistic software). The IC_50_ values were derived from SPSS nonlinear regression analysis.

### 3.3. Analysis of Cell Cycle by Flow Cytometry

The cell cycle was analyzed by flow cytometry. Firstly, HCT116 cells were treated with different concentrations of compound **4g** (0 μM, 1.56 μM, 3.125 μM, 6.25 μM, 12.5 μM, and 25 μM) for 72 h. After incubation, a total of (1–5) ×10^5^ cells were harvested from the treated and normal samples. The cells were washed twice with PBS and fixed in 70% ice-cold ethanol at least overnight. The sample was concentrated by removing the ethanol and then cells were then washed three times with PBS, staining the cellular DNA with fluorescent solution (1% (*v*/*v*) Triton X-100, 0.01% RNase, 0.05% PI) for 15 min in darkness. The cell cycle distribution was then detected by flow cytometry (COULTER EPICS XL, Orange City, FL, USA). All experiments were performed three times.

### 3.4. Parameter Prediction of Drugs

Adenomatous Polyposis Coli (APC) and Guanine Nucleate Exchange Factor (Asef) are proteins closely related to the occurrence and development of colon cancer. Clinical studies found that over 85% congenital colon cancer patients and 80% acquired colon cancer patients experience frameshift or deletion mutations in the APC gene, leading to the production of pathological APC proteins [52]. The pathological APC protein binds to the Asef, activating the Asef, which was originally suppressed by its own function, thereby promoting the carcinogenesis, tissue growth, and metastasis of colon cancer. With the deepening of scientific research on APC–Asef protein interactions, inhibiting this interactions can effectively inhibit the proliferation and metastasis of colon cancer cells. This conclusion has been accepted by researchers and has become one of “hotspots” in anti-colon cancer research [53,54,55]. The binding between pathological APC and Asef is essentially a protein–protein interaction (PPI) process, which has strong hydrophobicity at the interface and generally contains 56% non-polar groups, 29% polar groups, and 15% charged groups [56]. Additionally, the small molecule PPIs through virtual screening technology tend to be rich in aromatic structures [57]. Compared with traditional small-molecule drugs (200~500 Da), compounds acting on PPIs must be of higher molecular weight (>500 Da), larger in size (spatial volume), have stronger hydrophobicity, have more hydrogen bond receptors and ligands, and contain at least 4 rings, making it difficult to meet the Lipinski’s “rule of five”. The active derivatives we designed and synthesized using the molecular hybridization strategy had large molecular weights and volumes, strong hydrophobicity, and multiple ring systems. Moreover, the derivatives are mostly planar and can be inserted into the interface of APC and Asef, which is consistent with the properties of small molecule PPIs. The Molinspiration online prediction tool (https://www.molinspiration.com/, Slovenský Gr) was utilized to predict the molecular properties of the synthesized derivatives, such as the lipid water partition coefficient (miLogP), hydrogen bond acceptor (noN), hydrogen bond donor (nOHNH), molecular volume (MV), number of rotatable bonds (nrotb), and size of polar spatial region (TPSA) to evaluate the similarity between derivatives and PPIs.

### 3.5. Docking Study

Analysis of the structural domains of APC and Asef proteins reveals that hydrophobic amino acid residues dominate. The “hot spot” amino acid residue region is ^176^SHPGGGGEQLAINELISDG^194^ [58], with hydrophobic amino acid residues accounting for 58%. The active derivative belongs to polyphenolic compounds with multiple ring systems and strong hydrophobicity. The highly hydrophobic nature of protein receptors and small molecule ligands facilitates the formation of hydrophobic–hydrophobic interactions, resulting in a high affinity. We conducted molecular docking studies on the most potent compound **4g** with an APC–Asef crystal protein to observe whether it can block APC or Asef binding. 

Preparation for protein and small molecule calculations: Use Discovery Studio 4.5 software to analyze the complex protein crystals of APC–Asef binding in pathological types (PDB ID: 3NMX). Use ChemBioDraw Ultra 14.0 to draw the 2D structure of the ligand small molecule (**4g**) to be calculated, and then use the MMFF94 function in the 3D software module of ChemBioDraw Ultra 14.0 to perform initial energy optimization and structural stretching and save the data in a 3000 mol file format. 

Receptor ligand molecular docking: Using the Prepare protein function located in the Macroolecule module of DS, prepare and analyze protein receptors for PDB ID 3NMX protein crystals, including environmental cleavage, fragment repair, energy and conformation optimization, and action analysis. Analyze the protein binding region of APC–Asef in 3NMX protein, identify the physical binding center of the two, and delineate the binding site of the receptor protein lesion type APC to be calculated using the Define and Edit Binding Site function in the Receiver ligand interaction module, which is 1.5 times the size of the ligand molecule to be calculated. Import the previously prepared 3000 mol ligand file into DS software and further optimize the conformation of the ligand to be calculated using the Ligand Minimizing function in the Small Molecule module of DS. Using the CDOCKER function in the Receiver ligand interaction module, set the receptor as the lesion type APC protein and the ligand as the conformational optimized quasi computational molecular group, perform receptor ligand molecular docking, and obtain multiple randomly oriented specific receptor ligand binding conformations. Based on the specific situation of receptor binding, select the conformation with the highest negative value in the parameter CDOCKER-InterACTION-ENERGY (lowest binding energy, most stable binding) as the optimal conformation for each ligand. Visualize and analyze receptor ligand binding using the Show2D/3D Structure Diagram function in DS.

## 4. Conclusions

New derivatives of tetrahydrocurcumin were synthesized using a click reaction. The mono alkylation of the phenolic group with propargyl bromide followed by the copper(I)-catalyzed azide alkyne cycloaddition (CuAAC) resulted in the formation of linked triazole moiety tetrahydrocurcumin derivatives. The synthesized tetrahydrocurcumin derivatives were tested for their anticancer activity against four cancer cell lines. One of the prepared compounds including g showed potent inhibitory activity against human colon carcinoma HCT-116, with an IC_50_ value of 1.09 ± 0.17 μM. Molecular docking was investigated for compound **4g**, and the APC–Asef combined protein showed hydrophobic and hydrogen-bounding interactions between them. As a result of the obtained anticancer activity, these compounds serve as important building blocks for future anticancer agents and can be further improved by further modification of tetrahydrocurcumin. The results obtained herein are important for further structure modifications of THC and the exploitation of the therapeutic potential of THC derivatives as anticancer agents. 

## Figures and Tables

**Table 1:** The anti-proliferative activity data (IC_50_) of the prepared compounds (*n* = 3).

Compound	IC_50_ (μM)			
	HCT116	HeLa	A549	HepG2
**Tetrahydrocurcumin**	50.96 ± 0.23	33.88 ± 0.35	69.25 ± 0.48	60.39 ± 0.19
**Cisplatin**	8.22 ± 0.86	10.12 ± 0.06	28.10 ± 1.58	98.22 ± 1.33
**4a**	62.63 ± 3.06	>200	>200	>200
**4b**	>200	>200	>200	>200
**4c**	>200	>200	>200	>200
**4d**	>200	>200	>200	>200
**4e**	>200	>200	162.40 ± 2.20	>200
**4f**	15.59 ± 0.45	>200	>200	53.64 ± 0.27
**4g**	1.09 ± 0.17	190 ± 0.67	45.16 ± 0.92	66.82 ± 0.42
**4h**	69.74 ± 7.12	>200	>200	>200
**4i**	>200	>200	>200	>200
**4j**	89.38 ± 1.39	175.9 ± 1.21	131.20 ± 1.00	>200
**4k**	72.07 ± 3.88	129.5 ± 3.46	57.96 ± 3.78	104.23 ± 0.23
**4l**	>200	>200	>200	>200
**4m**	>200	>200	135.60 ± 4.80	>200

**Table 2:** Parameter prediction of derivative drugs.

Compound	MW	Natoms	miLogP	nON	nOHNH	Nrotb	TPSA	MV
**4a**	529.59	39	3.58	9	1	14	112.79	482.19
**4b**	543.62	40	4.02	9	1	14	112.79	498.76
**4c**	557.65	41	4.49	9	1	15	112.79	515.56
**4d**	571.67	42	5.09	9	1	15	112.79	532.14
**4e**	585.70	43	5.28	9	1	15	112.79	548.38
**4f**	559.62	41	3.63	10	1	15	122.02	507.74
**4g**	547.58	40	3.74	9	1	14	112.79	547.58
**4h**	564.04	40	4.25	9	1	14	112.79	495.73
**4i**	608.49	40	4.38	9	1	14	112.79	500.08
**4j**	613.59	44	4.54	10	1	16	122.02	613.59
**4k**	579.59	43	4.47	9	1	15	112.79	513.49
**4l**	576.56	42	2.89	13	1	15	167.85	497.71
**4m**	554.60	41	3.33	10	1	14	136.58	499.06

## Data Availability

Data are contained within the article and Supplementary Materials.

## Supplementary Materials

The following supporting information can be downloaded at: https://www.mdpi.com/article/10.3390/molecules29133010/s1, Copies of the ^1^H NMR, ^13^C NMR, IR, and HRMS spectra for compounds **4a**–**4m**.
