# Supplementary material for: Synthesis, Anticancer Activity, and Molecular Docking of New 1,2,3-Triazole Linked Tetrahydrocurcumin Derivatives
Source: Molecules. 2024 Jun 25;29(13):3010. doi: 10.3390/molecules29133010 (PMC11243220; doi:10.3390/molecules29133010)
Supplement: Supplementary file 1 [file molecules-29-03010-s001.zip › molecules-2967202-supplementary.pdf]

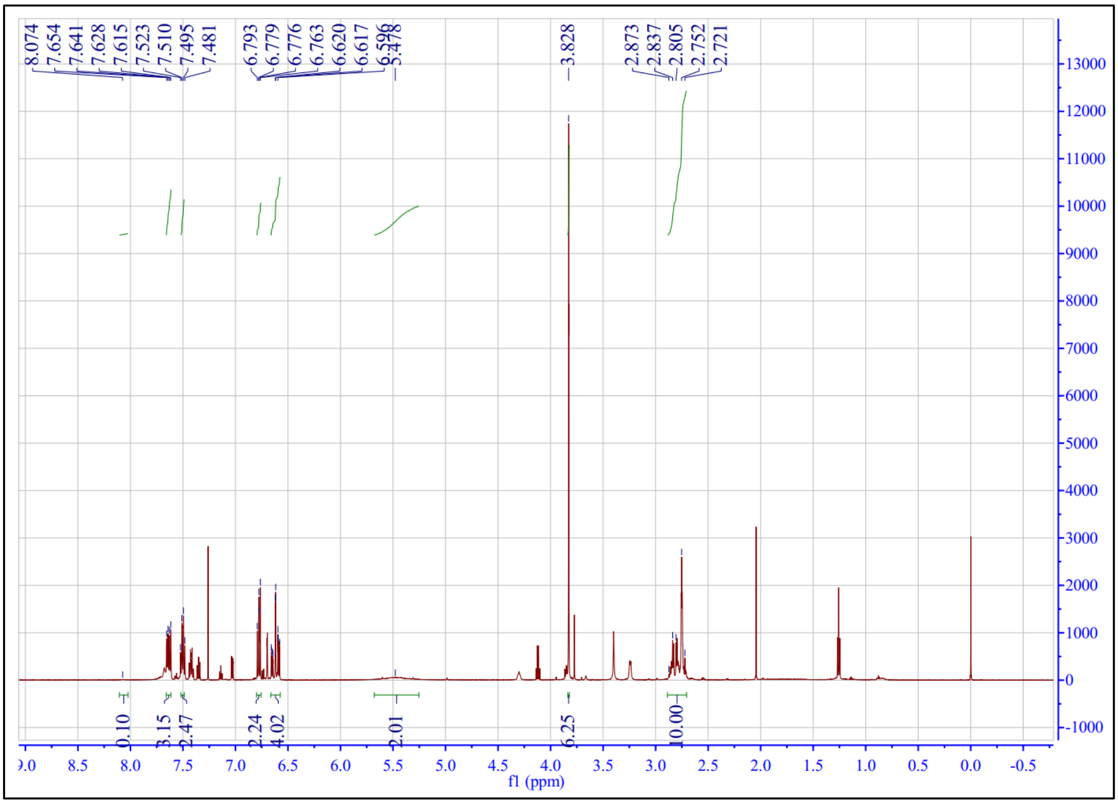

8.074
7.654
7.641
7.628
7.615
7.523
7.510
7.495
7.481
6.793
6.779
6.776
6.763
6.620
6.617
6.596
5.478
3.828
2.873
2.837
2.805
2.752
2.721
0.10
3.15
2.47
2.24
4.02
2.01
6.25
10.00
f1 (ppm)

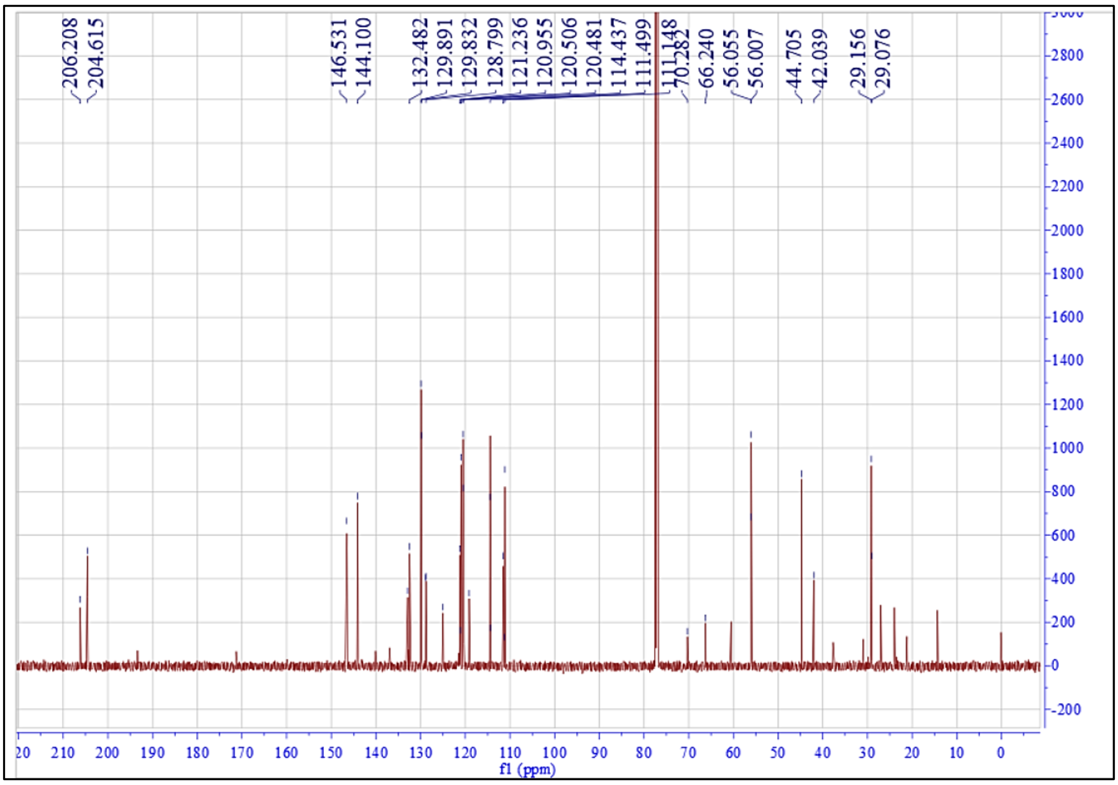

206.208
204.615
146.531
144.100
132.482
129.891
129.832
128.799
121.236
120.955
120.506
120.481
114.437
111.499
111.148
70.282
66.240
56.055
56.007
44.705
42.039
29.156
29.076
f1 (ppm)

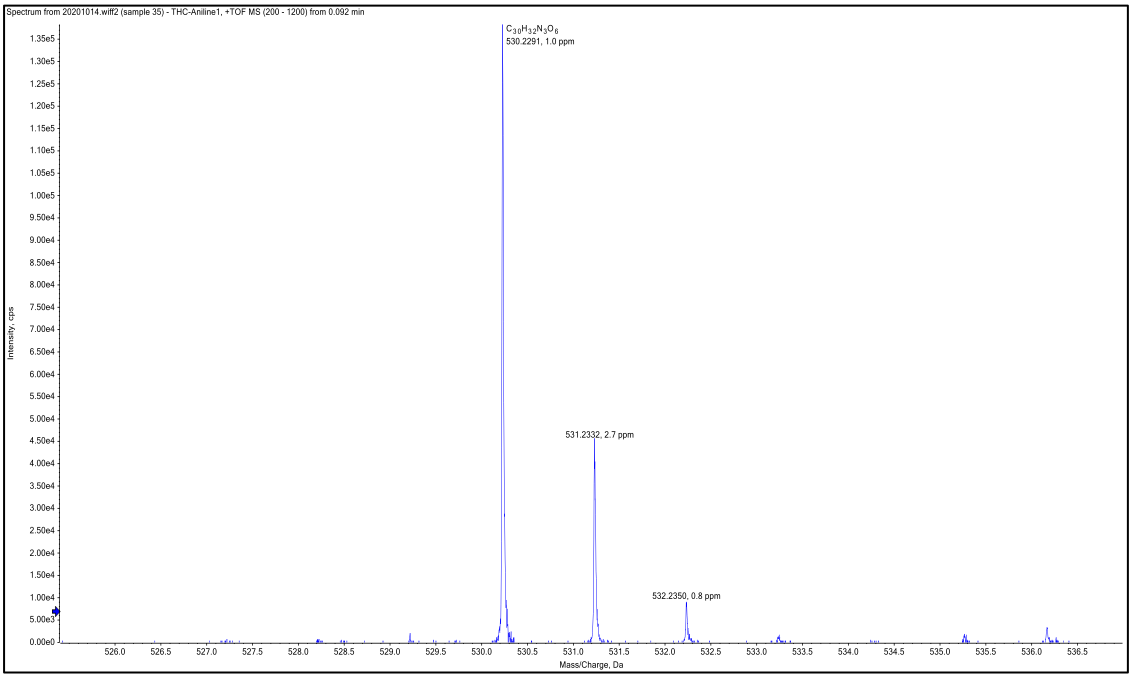

Figure S1. ^{1}H-NMR, ^{13}C-NMR, HR-ESI-MS spectrum of **4a**

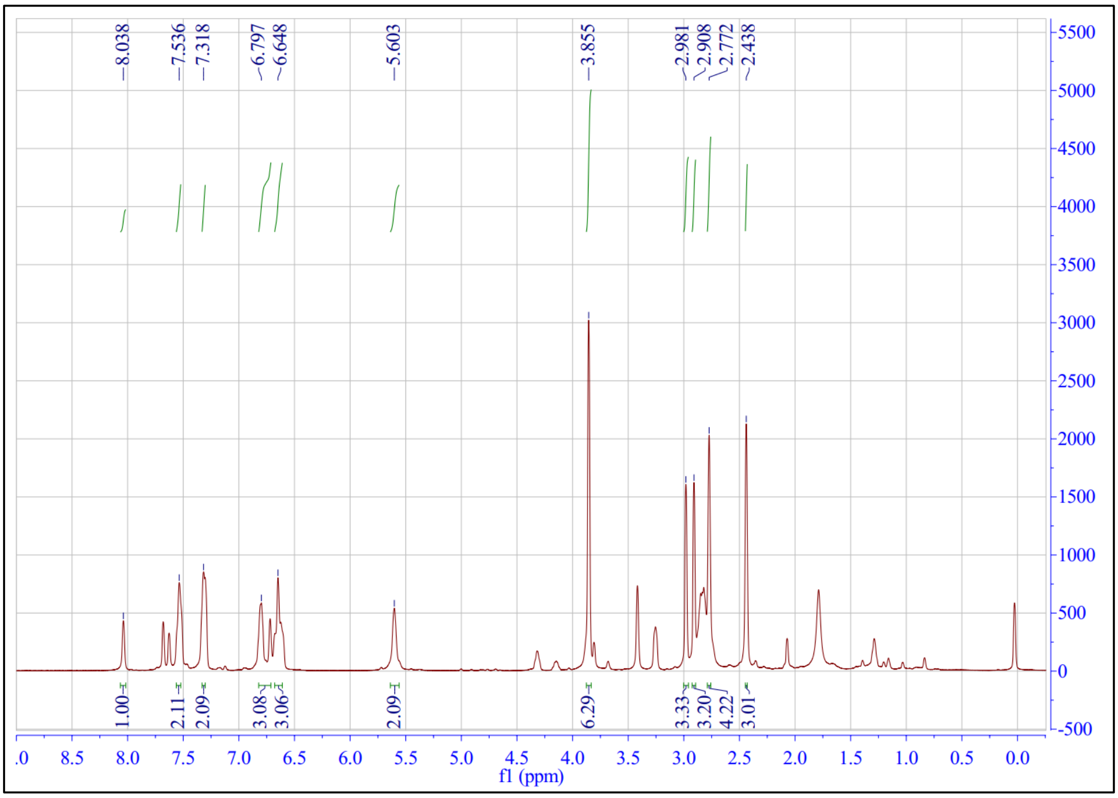

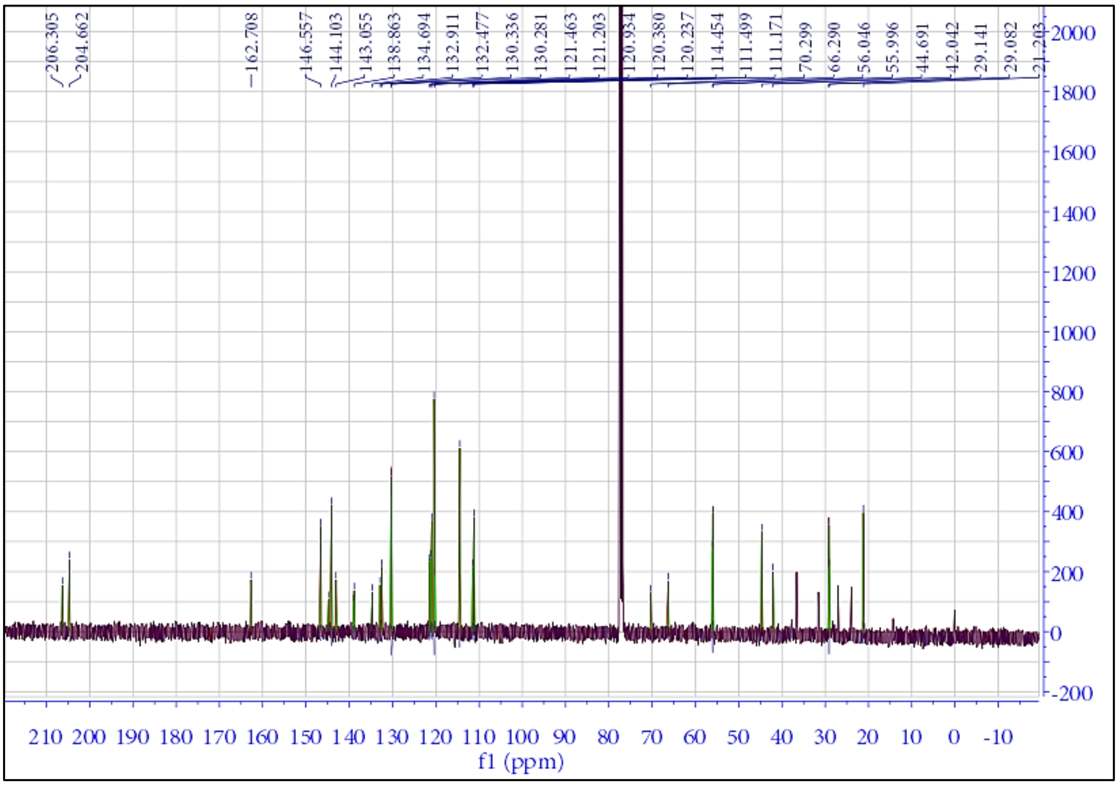

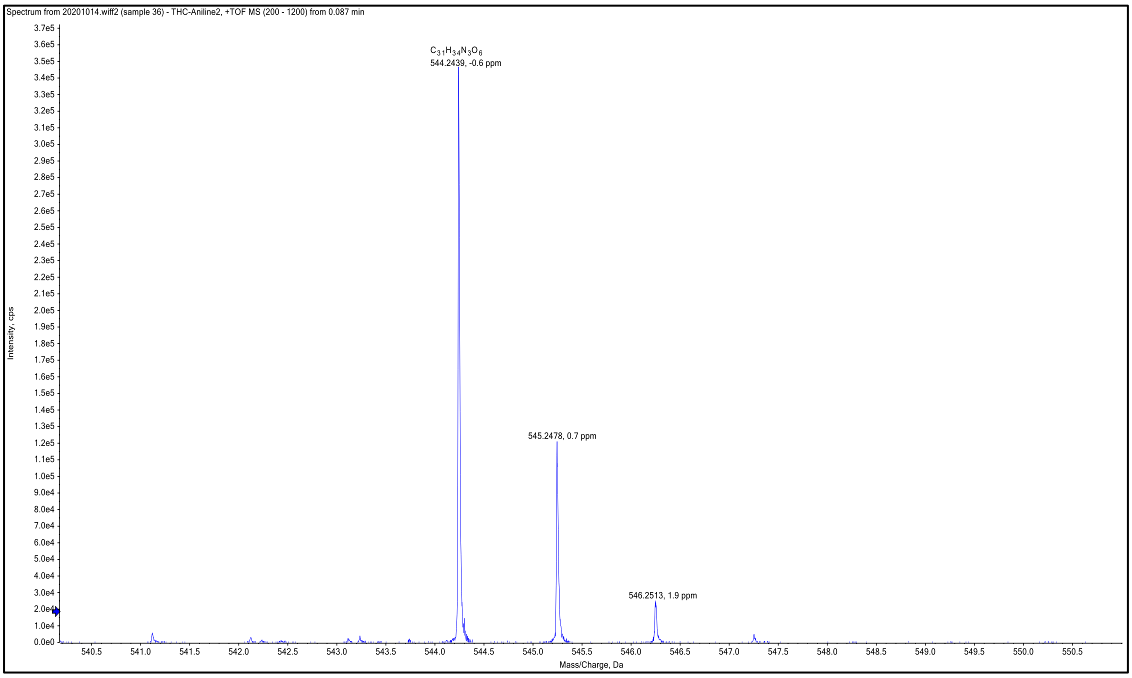

Figure S2. ^{1}H-NMR, ^{13}C-NMR, HR-ESI-MS spectrum of **4b**

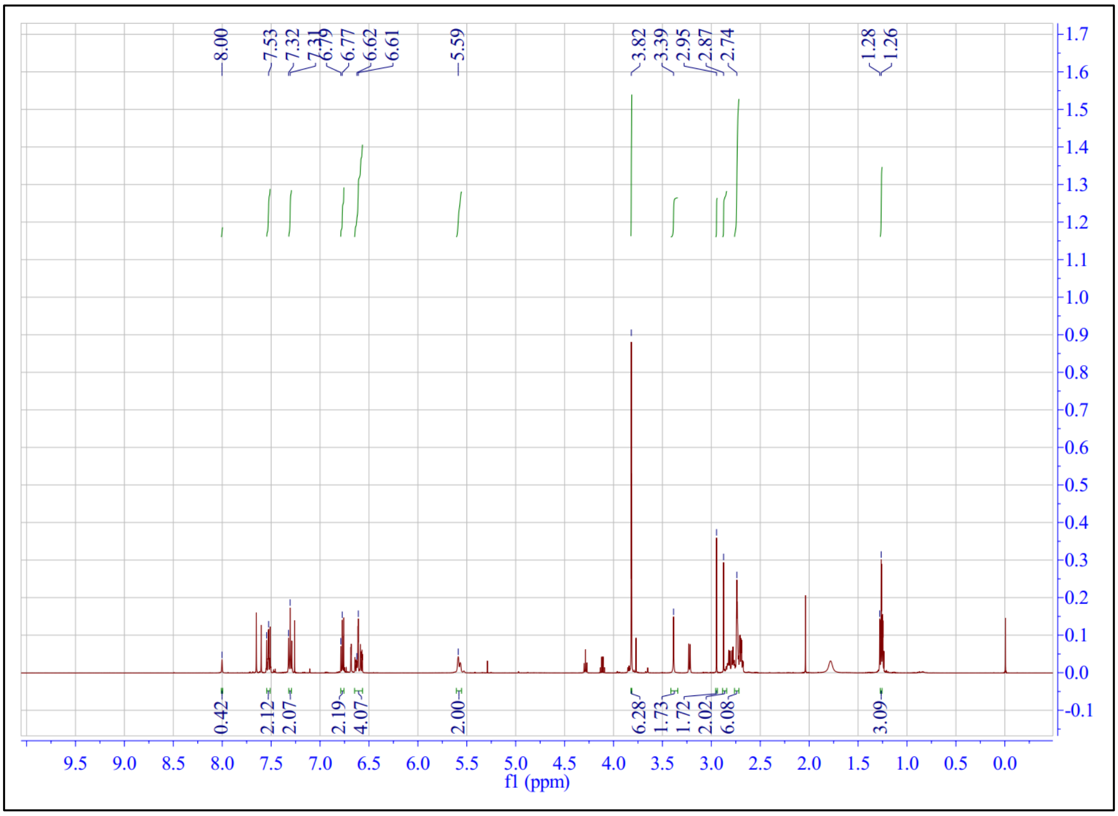

8.00
7.53
7.32
7.31
6.79
6.77
6.62
6.61
5.59
3.82
3.39
2.95
2.87
2.74
1.28
1.26
0.42
2.12
2.07
2.19
4.07
2.00
6.28
1.73
1.72
2.02
6.08
3.09
f1 (ppm)

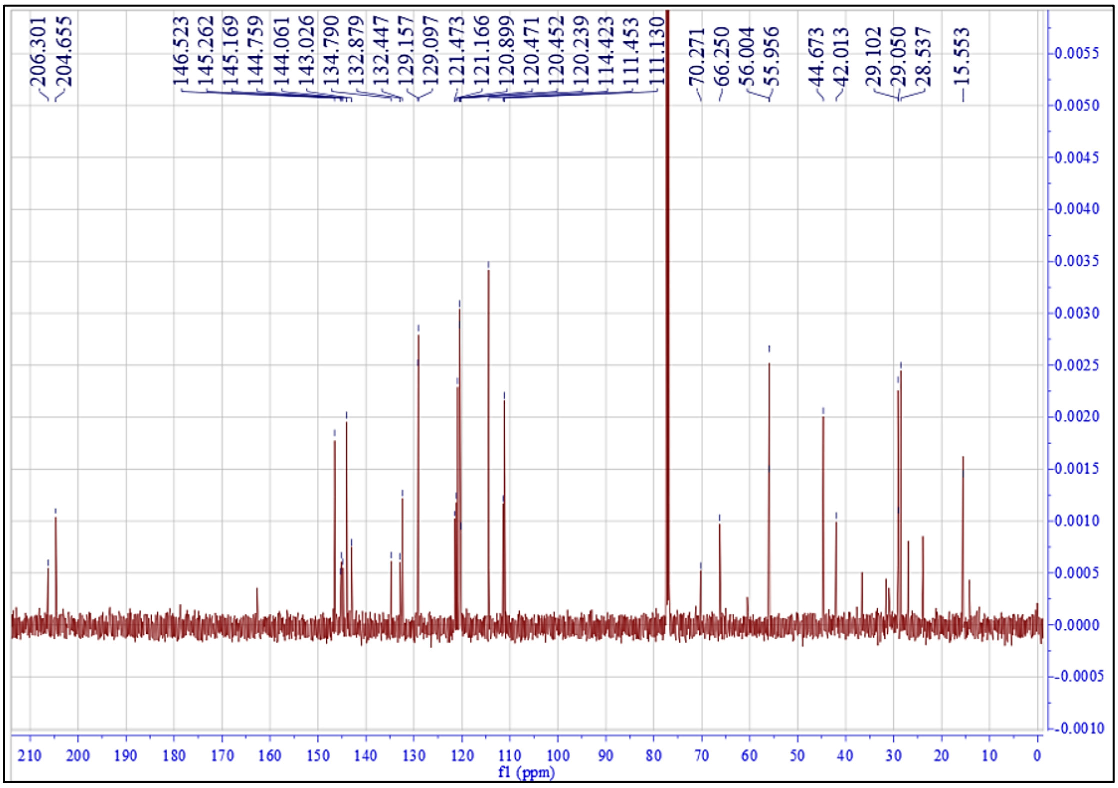

206.301
204.655
146.523
145.262
145.169
144.759
144.061
143.026
134.790
132.879
132.447
129.157
129.097
121.473
121.166
120.899
120.471
120.452
120.239
114.423
111.453
111.130
70.271
66.250
56.004
55.956
44.673
42.013
29.102
29.050
28.537
15.553
f1 (ppm)

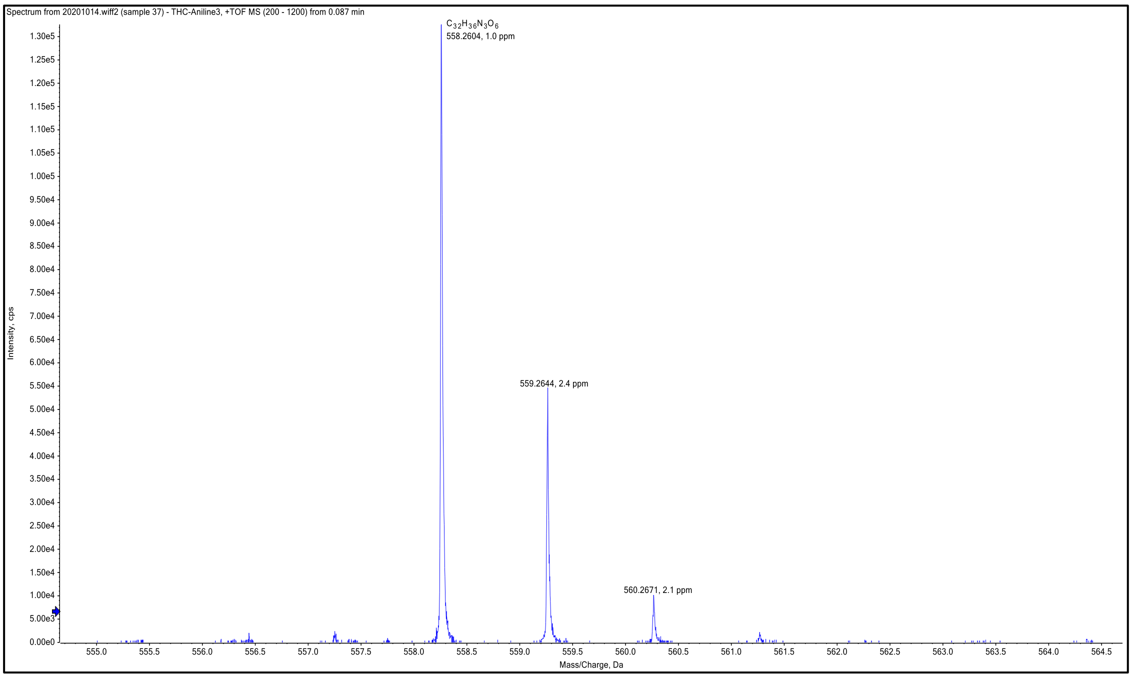

Figure S3. ^{1}H-NMR, ^{13}C-NMR, HR-ESI-MS spectrum of **4c**

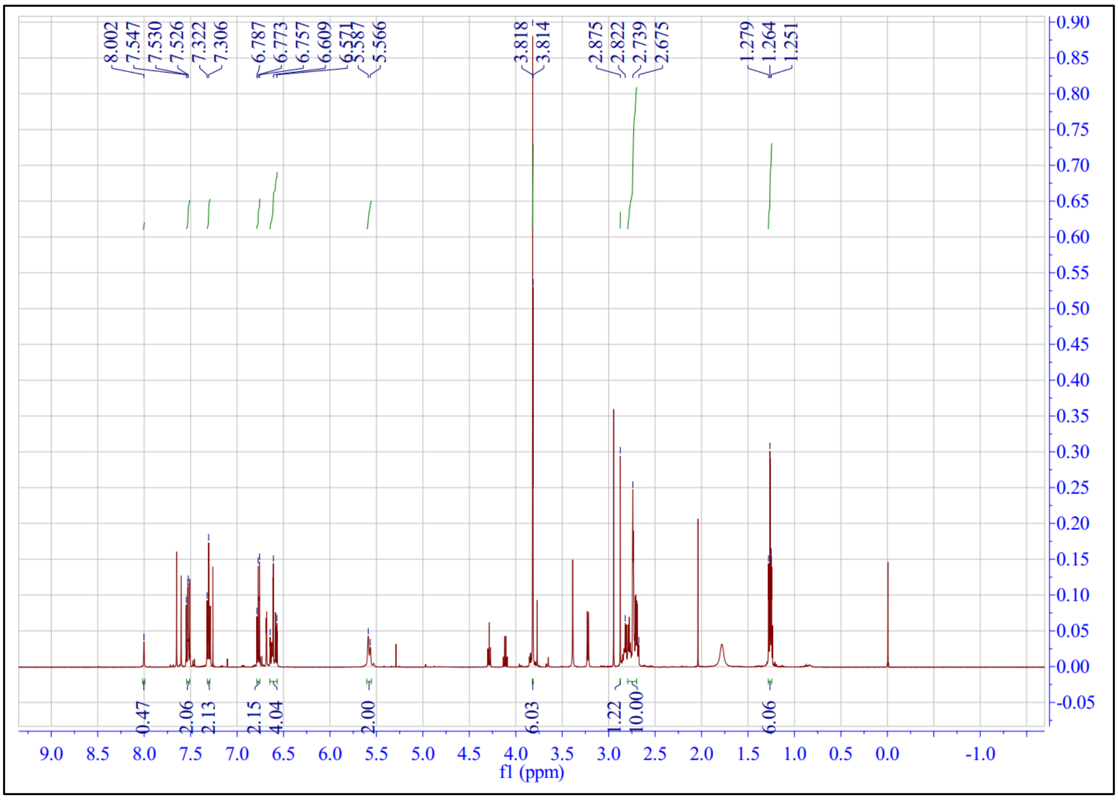

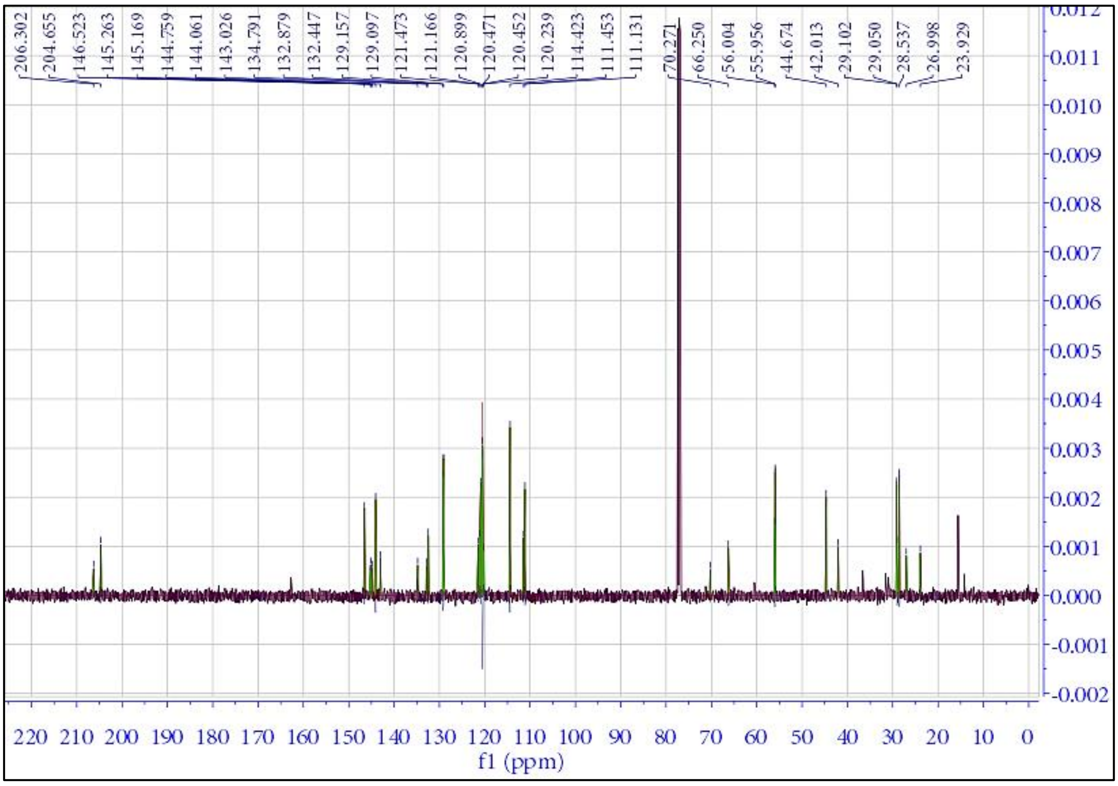

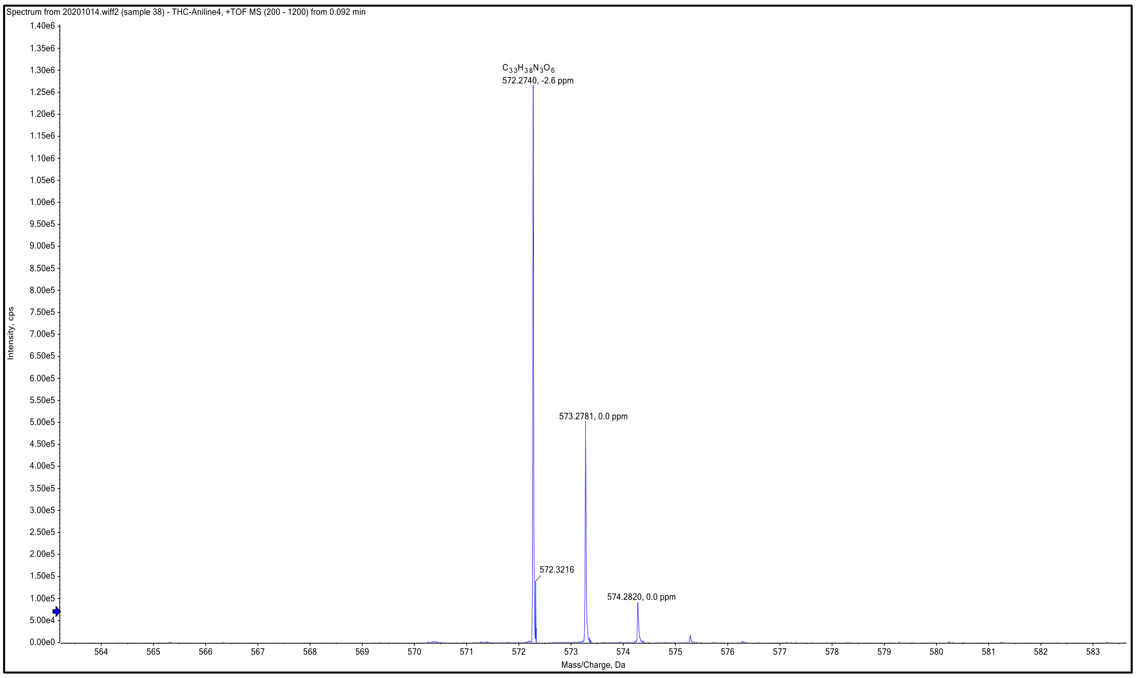

Figure S4. ^{1}H-NMR, ^{13}C-NMR, HR-ESI-MS spectrum of **4d**

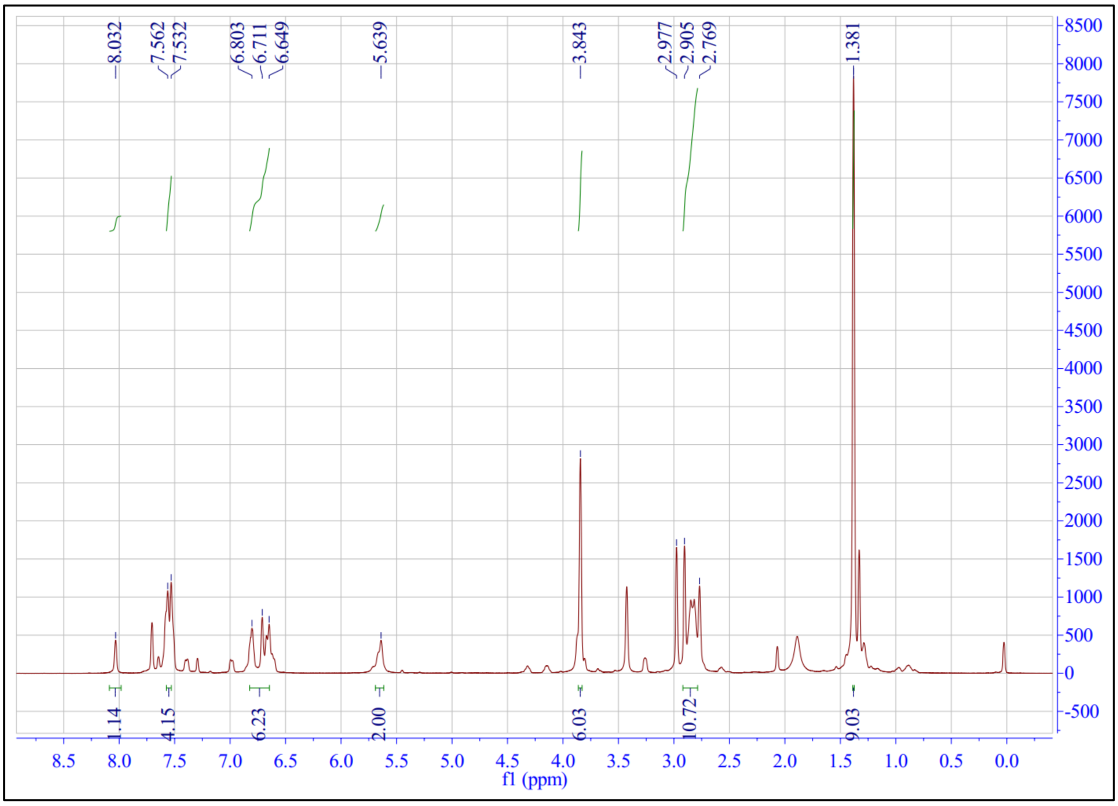
8.032
7.562
7.532
6.803
6.711
6.649
5.639
3.843
2.977
2.905
2.769
1.381
1.14
4.15
6.23
2.00
6.03
10.72
9.03
f1 (ppm)

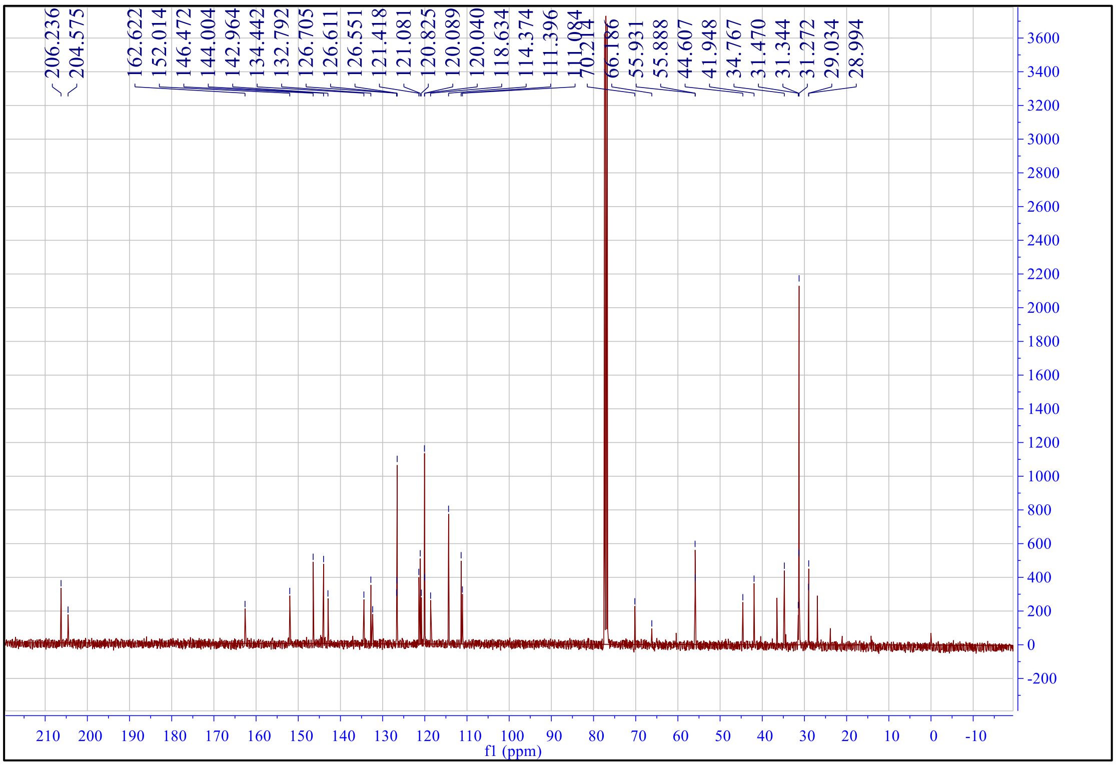
206.236
204.575
162.622
152.014
146.472
144.004
142.964
134.442
132.792
126.705
126.611
126.551
121.418
121.081
120.825
120.089
120.040
118.634
114.374
111.396
111.084
70.214
66.186
55.931
55.888
44.607
41.948
34.767
31.470
31.344
31.272
29.034
28.994
f1 (ppm)

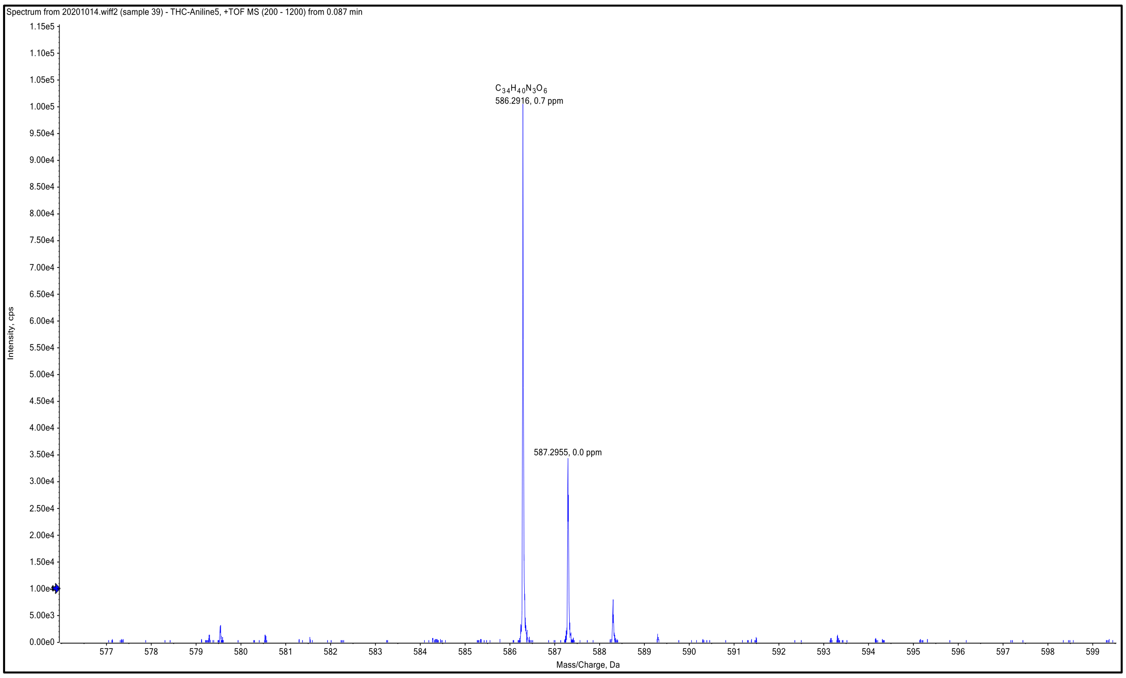

Figure S5. ^{1}H-NMR, ^{13}C-NMR, HR-ESI-MS spectrum of **4e**

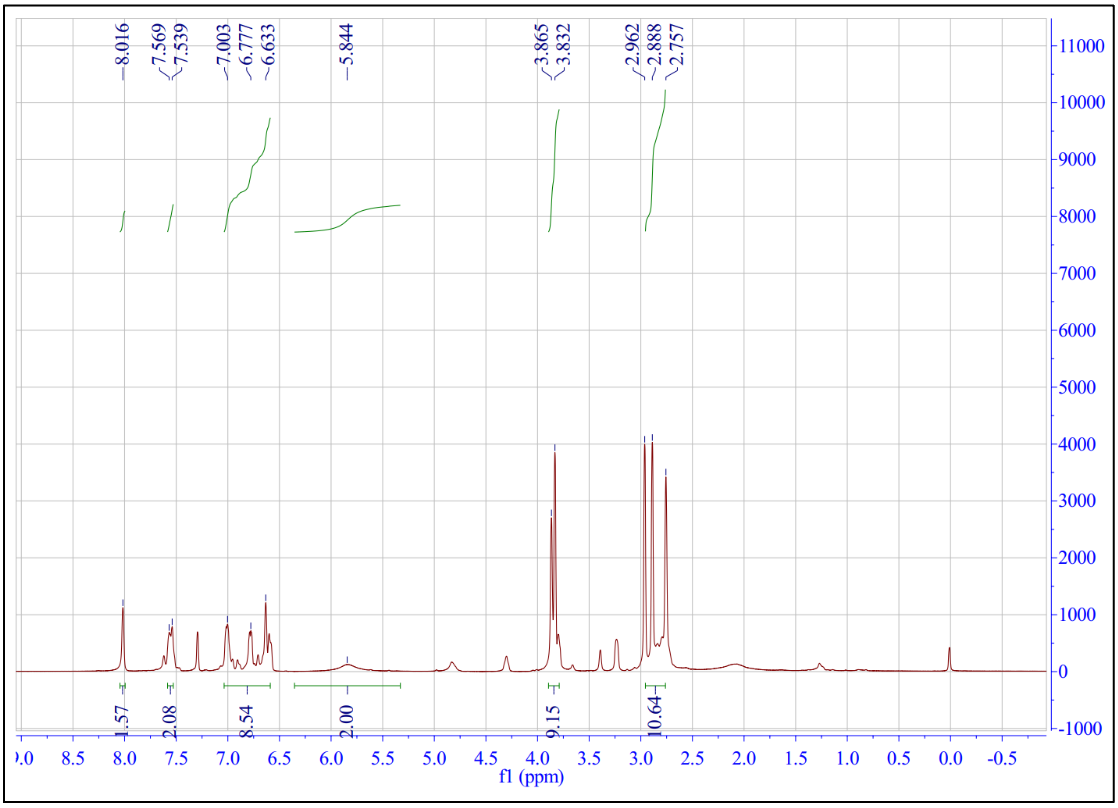

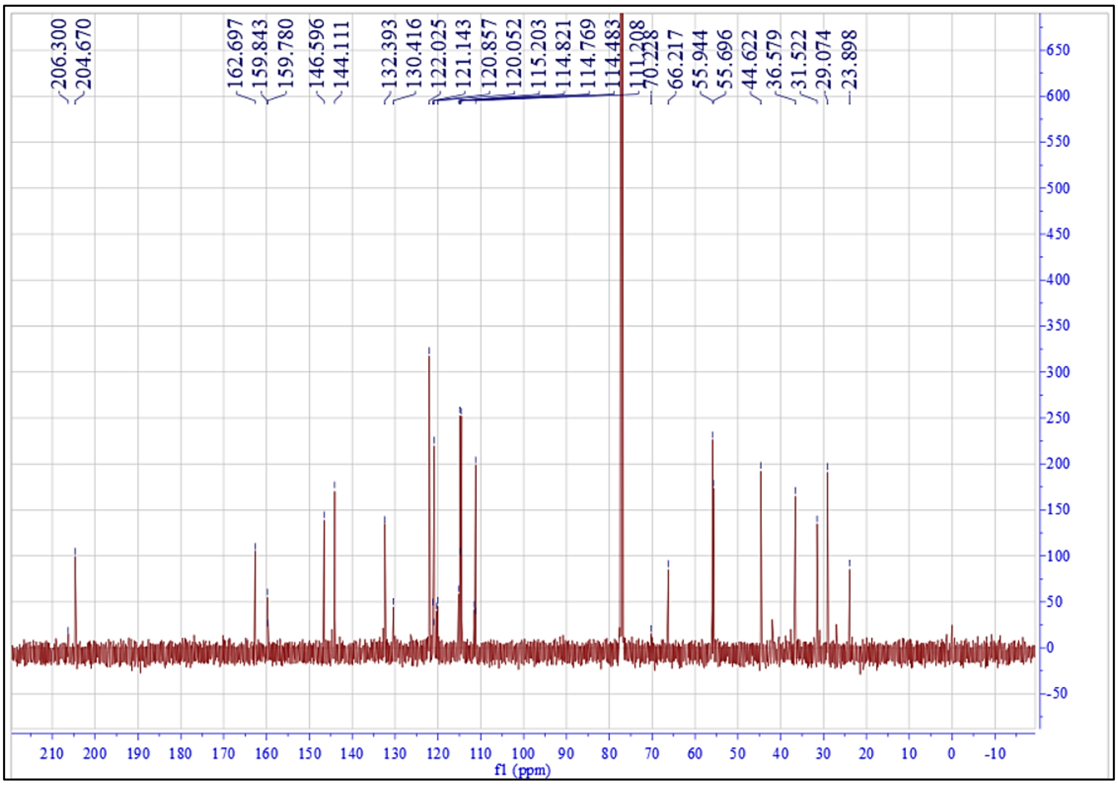

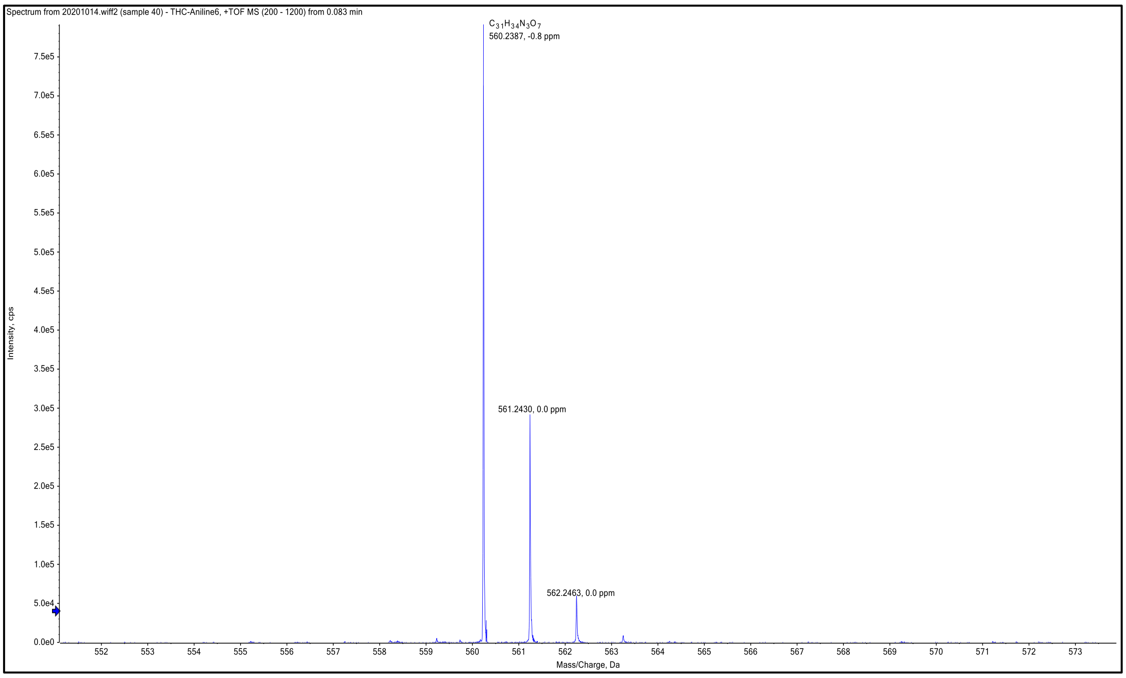

Figure S6. ^{1}H-NMR, ^{13}C-NMR, HR-ESI-MS spectrum of **4f**

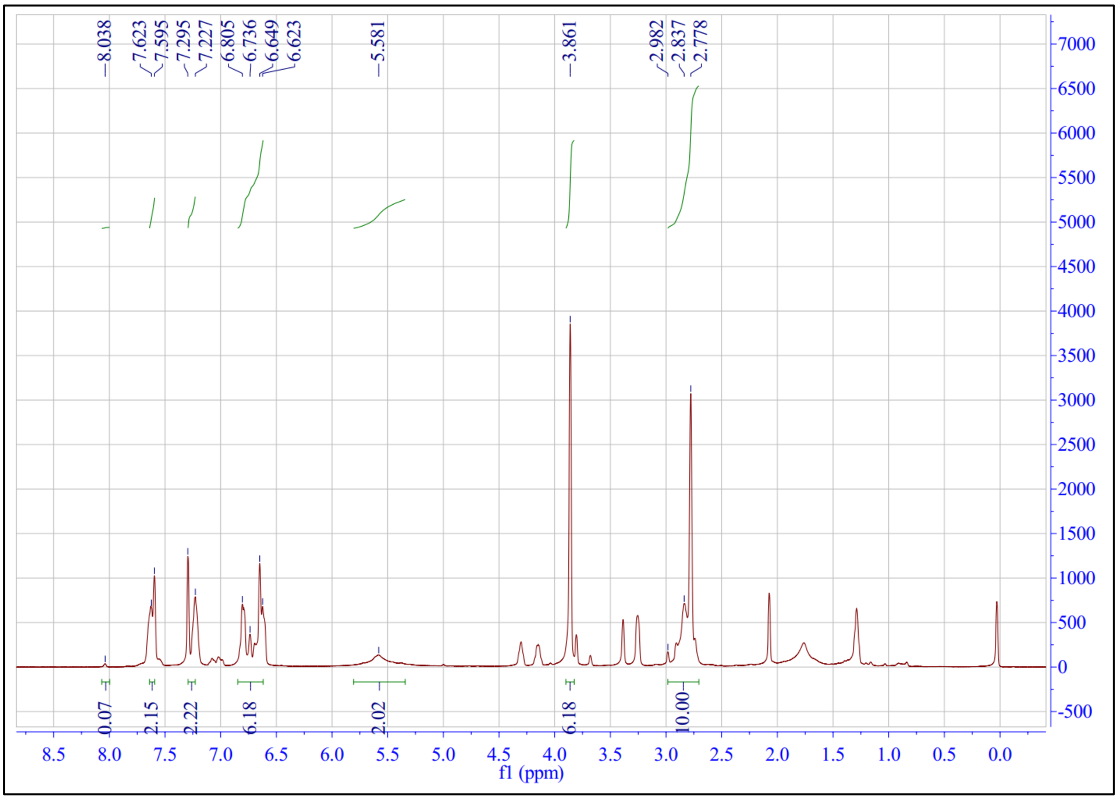

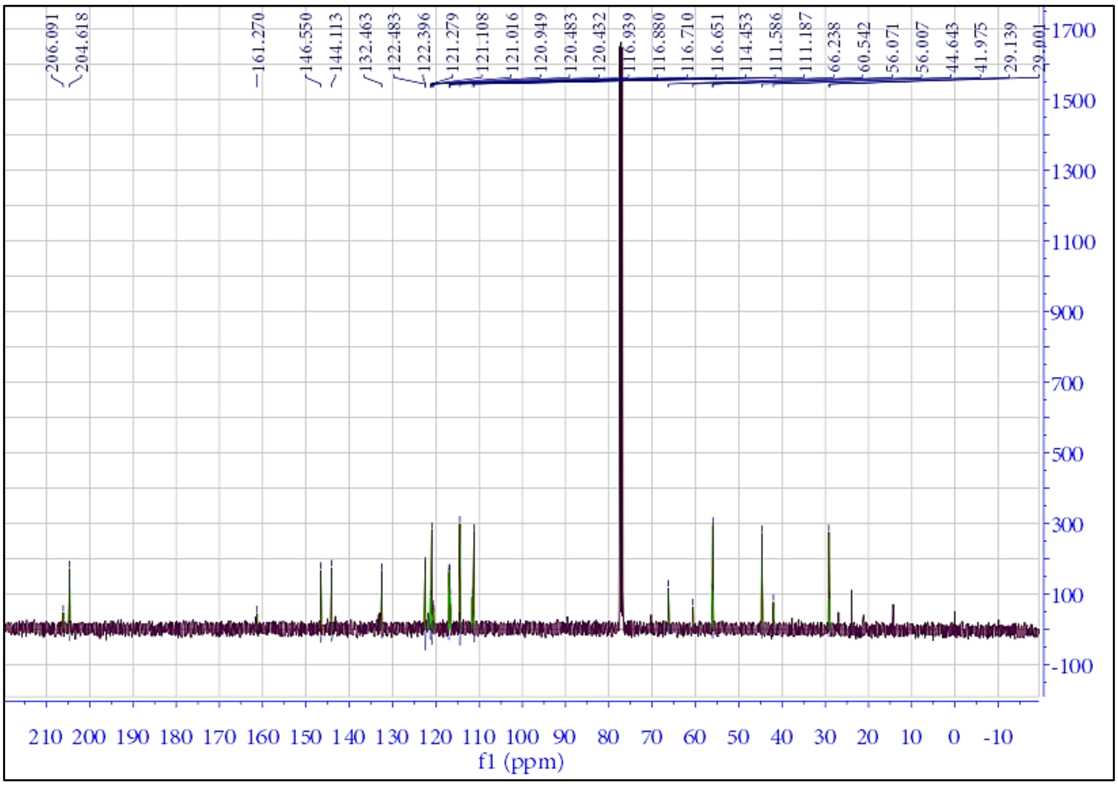

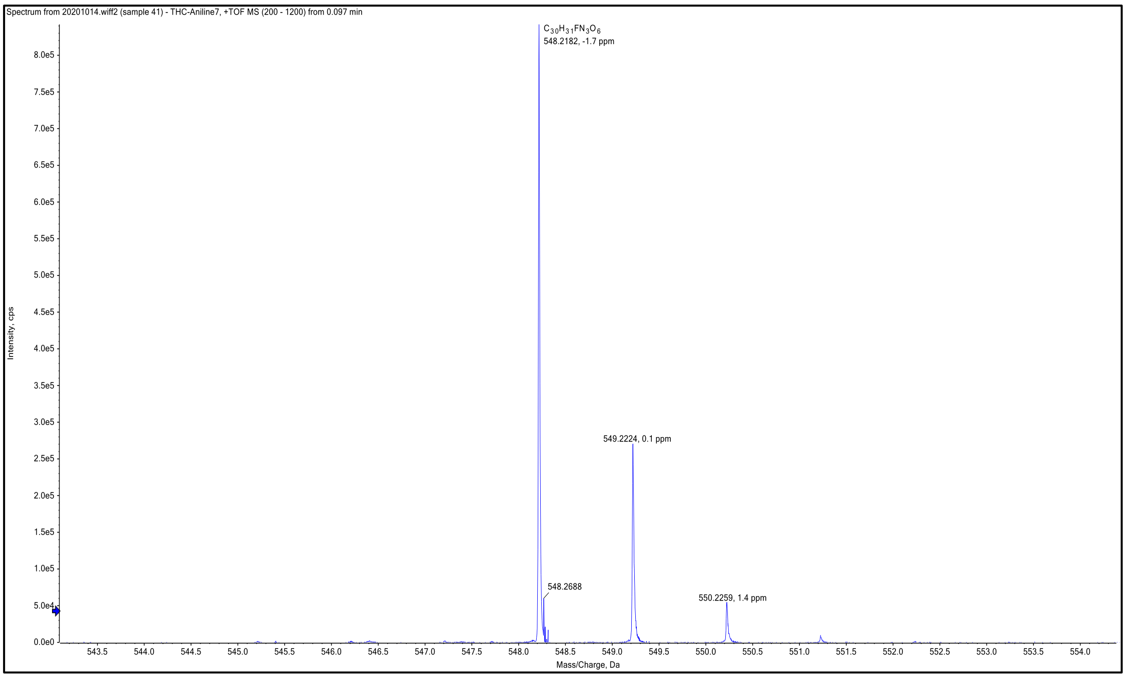

Figure S7. ^{1}H-NMR, ^{13}C-NMR, HR-ESI-MS spectrum of **4g**

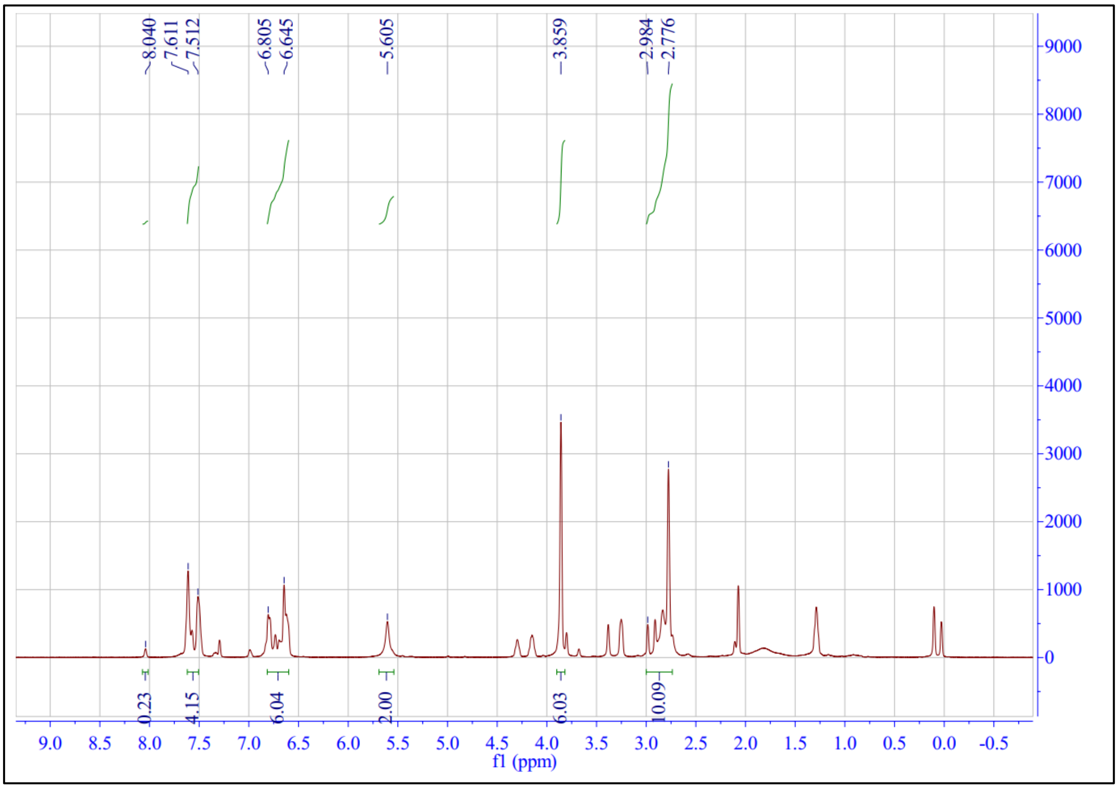

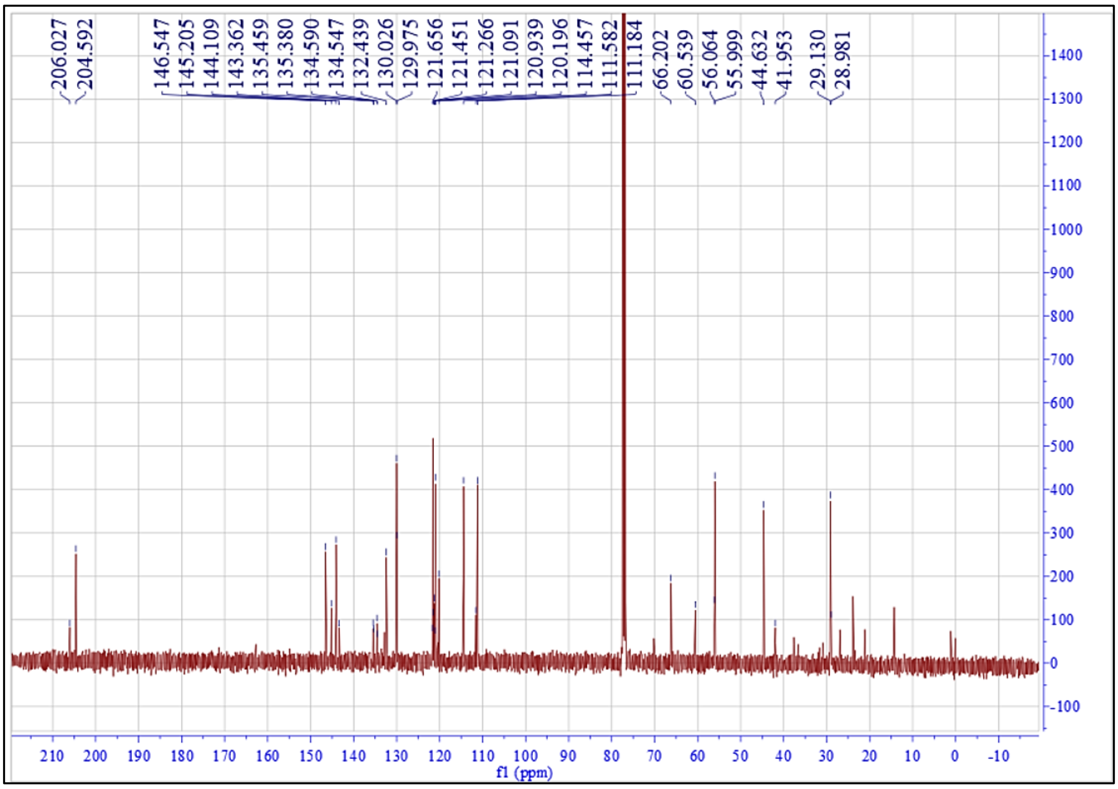

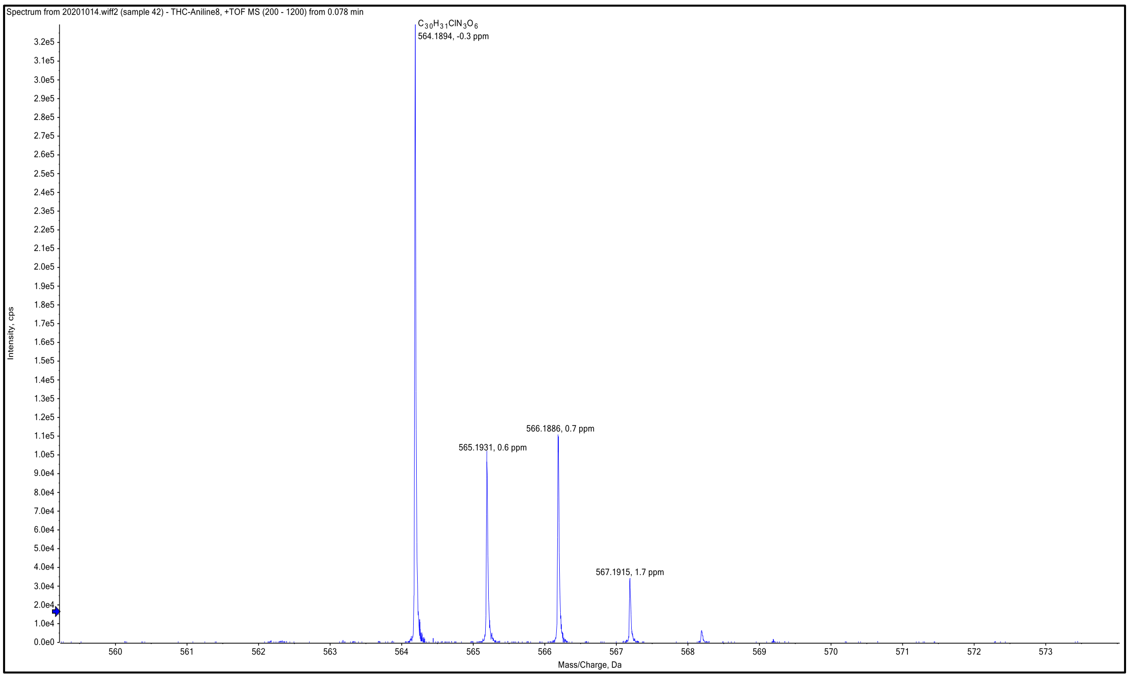

Figure S8. ^{1}H-NMR, ^{13}C-NMR, HR-ESI-MS spectrum of **4h**

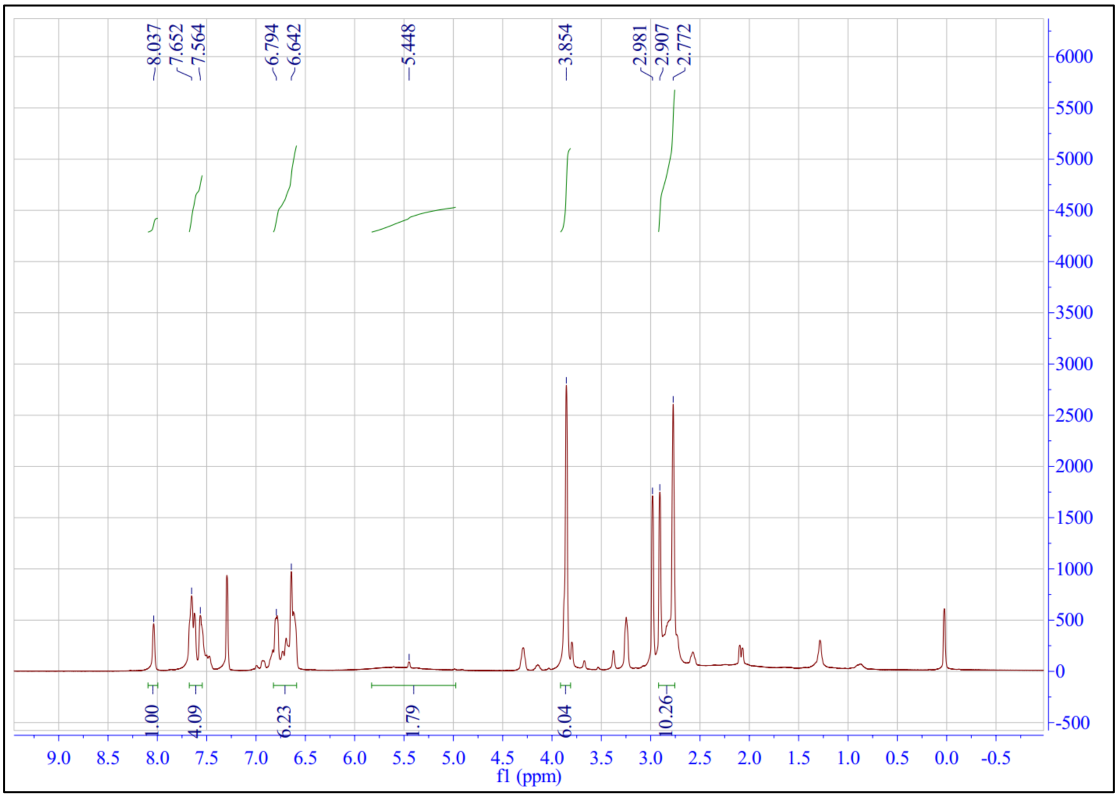
8.037
7.652
7.564
6.794
6.642
5.448
3.854
2.981
2.907
2.772
1.00
4.09
6.23
1.79
6.04
10.26
9.0 8.5 8.0 7.5 7.0 6.5 6.0 5.5 5.0 4.5 4.0 3.5 3.0 2.5 2.0 1.5 1.0 0.5 0.0 -0.5
f1 (ppm)
6000 5500 5000 4500 4000 3500 3000 2500 2000 1500 1000 500 0 -500

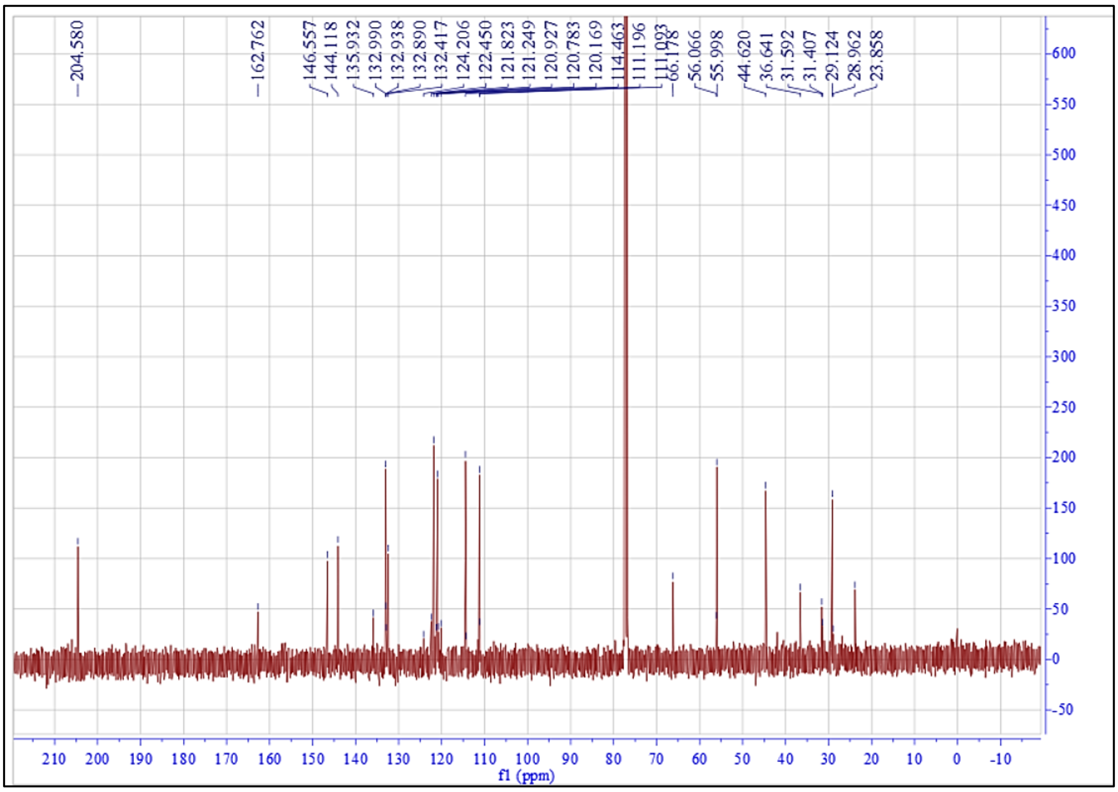
204.580
162.762
146.557
144.118
135.932
132.990
132.938
132.890
132.417
124.206
122.450
121.823
121.249
120.927
120.783
120.169
114.463
111.196
111.093
66.178
56.066
55.998
44.620
36.641
31.592
31.407
29.124
28.962
23.858
210 200 190 180 170 160 150 140 130 120 110 100 90 80 70 60 50 40 30 20 10 0 -10
f1 (ppm)
600 550 500 450 400 350 300 250 200 150 100 50 0 -50

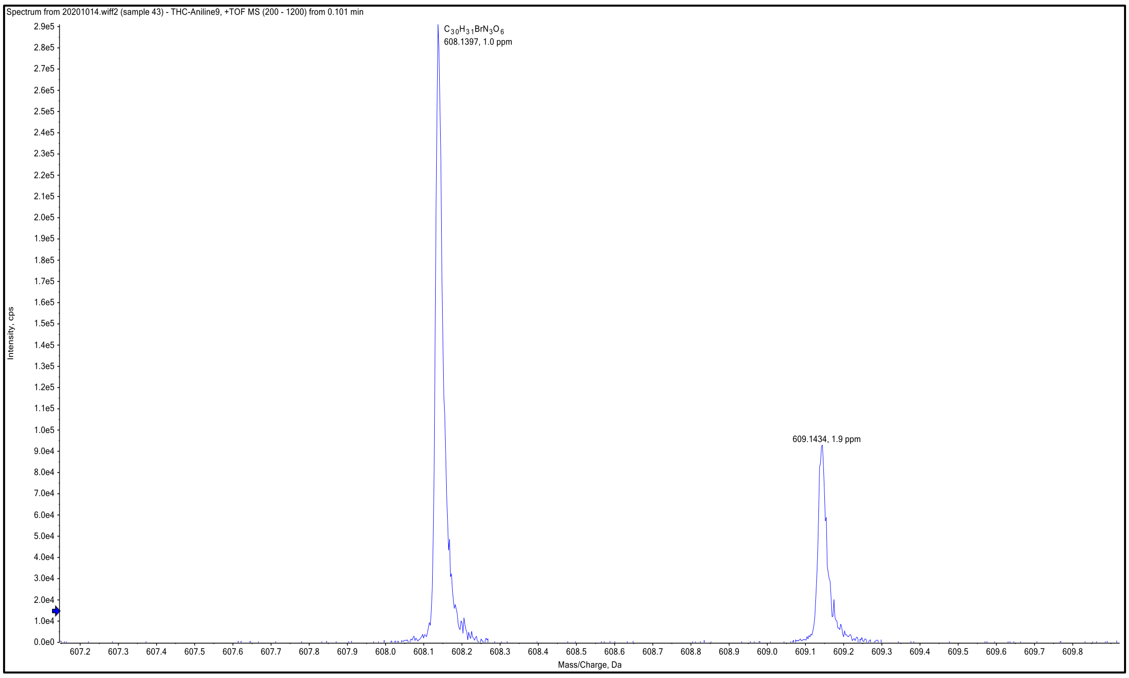

Figure S9. ^{1}H-NMR, ^{13}C-NMR, HR-ESI-MS spectrum of **4i**

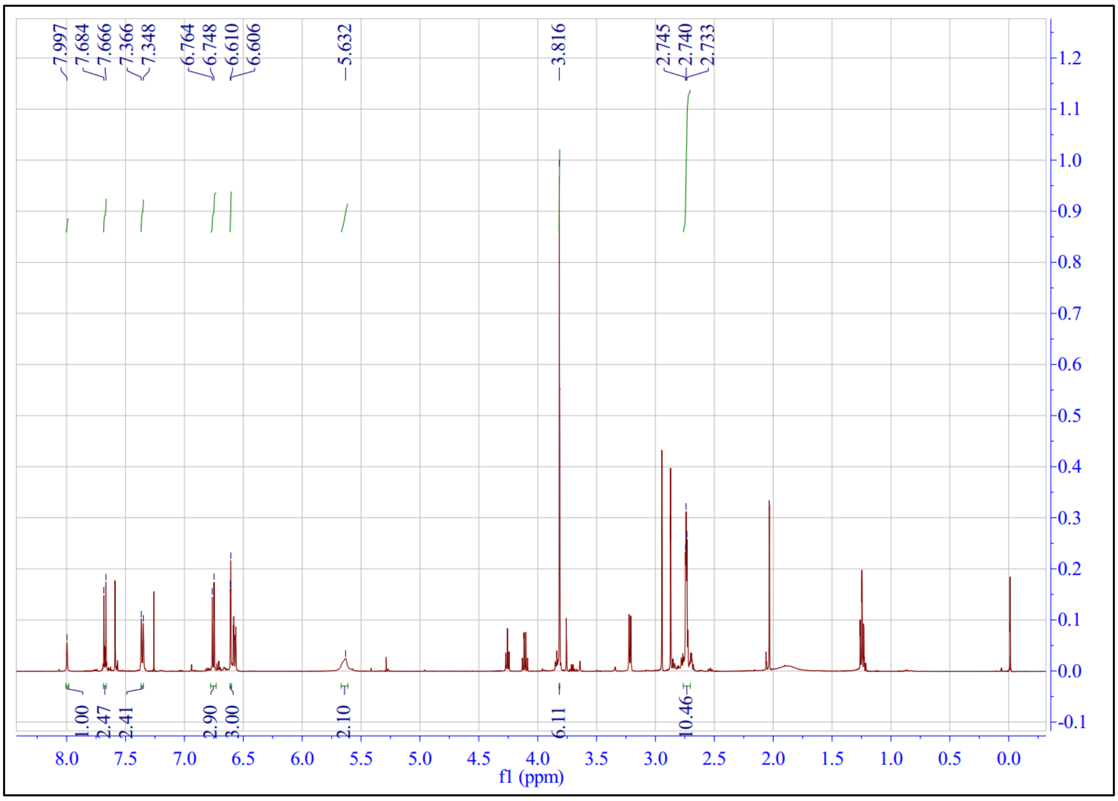

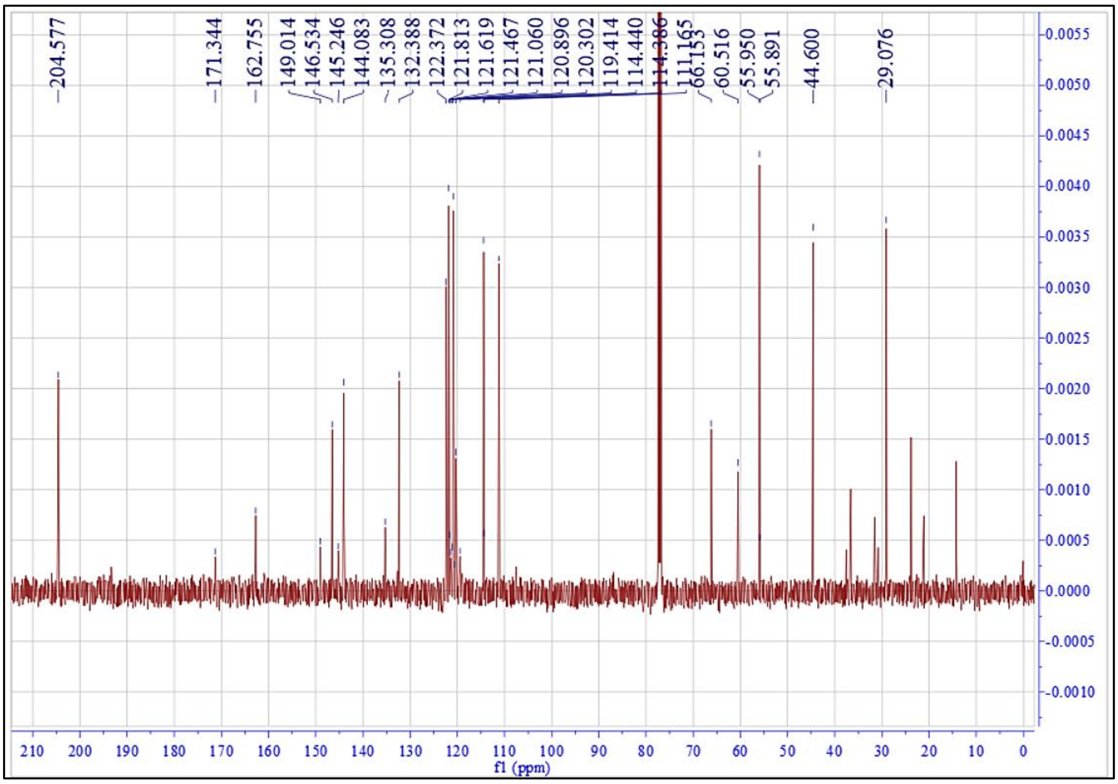

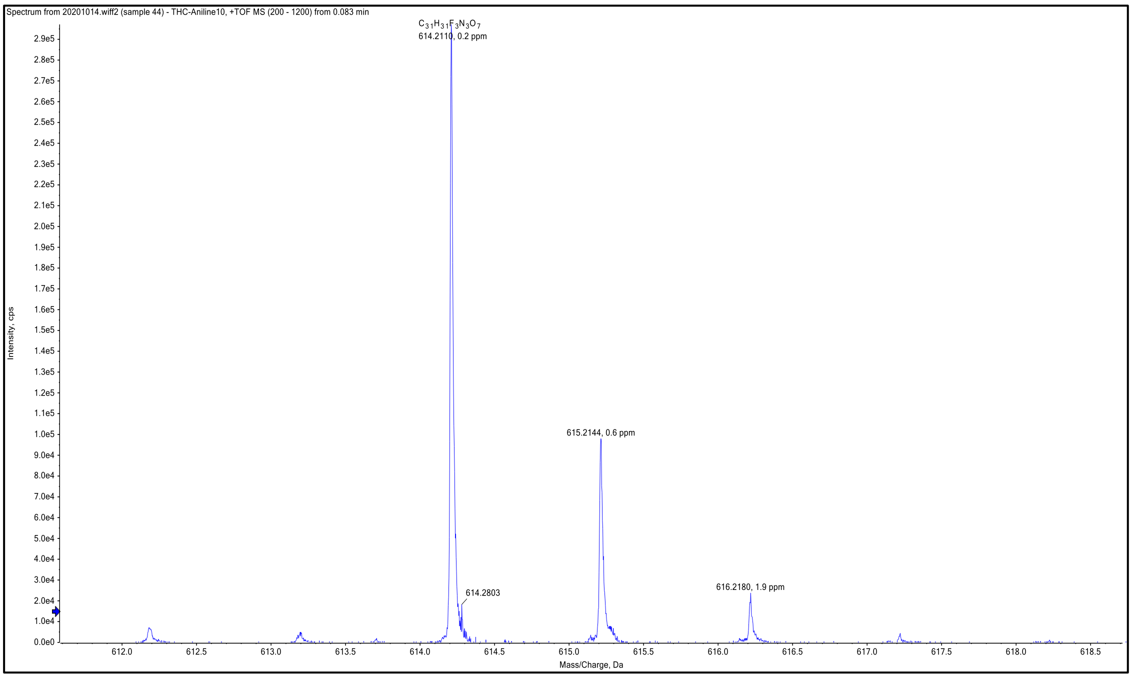

Figure S10. ^{1}H-NMR, ^{13}C-NMR, HR-ESI-MS spectrum of **4j**

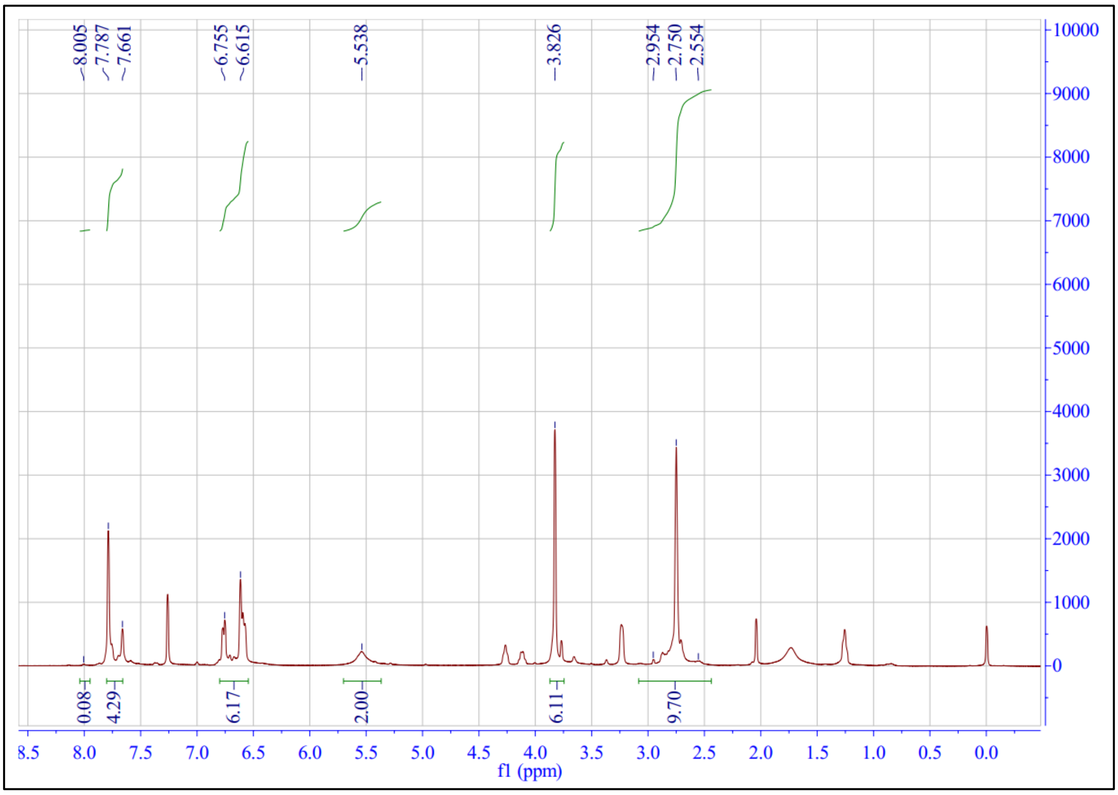
8.005
7.787
7.661
6.755
6.615
5.538
3.826
2.954
2.750
2.554
0.08
4.29
6.17
2.00
6.11
9.70
f1 (ppm)

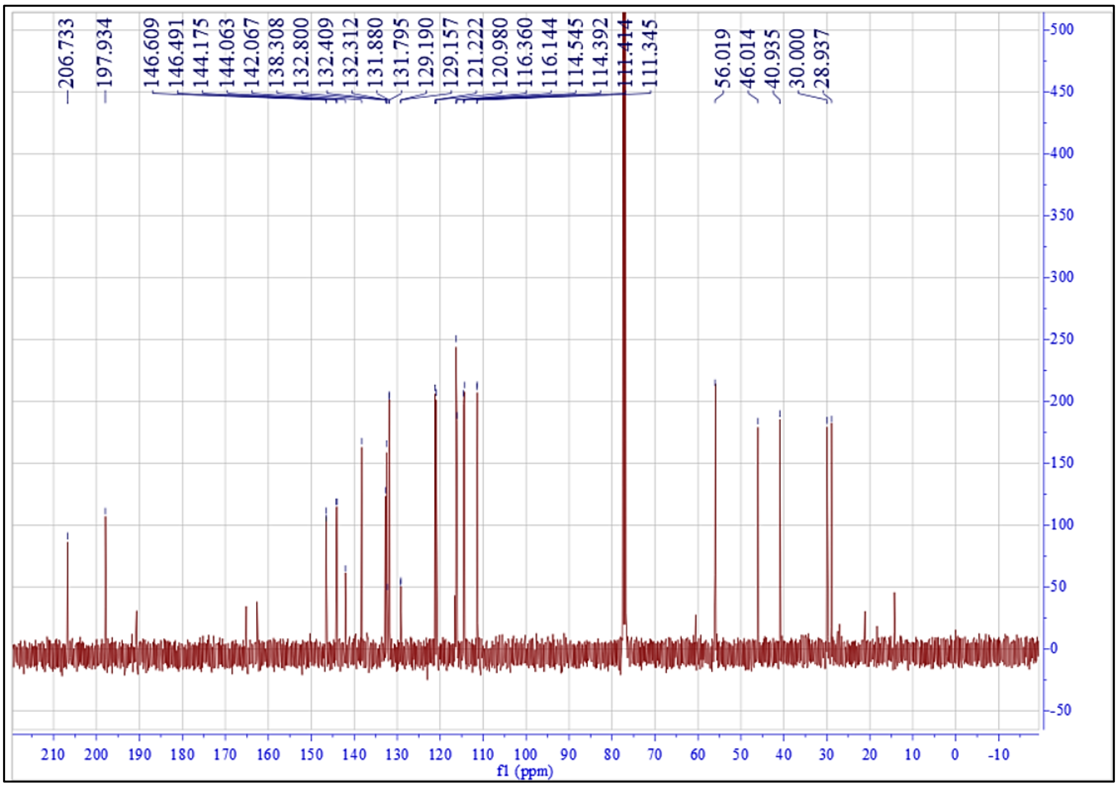
206.733
197.934
146.609
146.491
144.175
144.063
142.067
138.308
132.800
132.409
132.312
131.880
131.795
129.190
129.157
121.222
120.980
116.360
116.144
114.545
114.392
111.414
111.345
56.019
46.014
40.935
30.000
28.937
f1 (ppm)

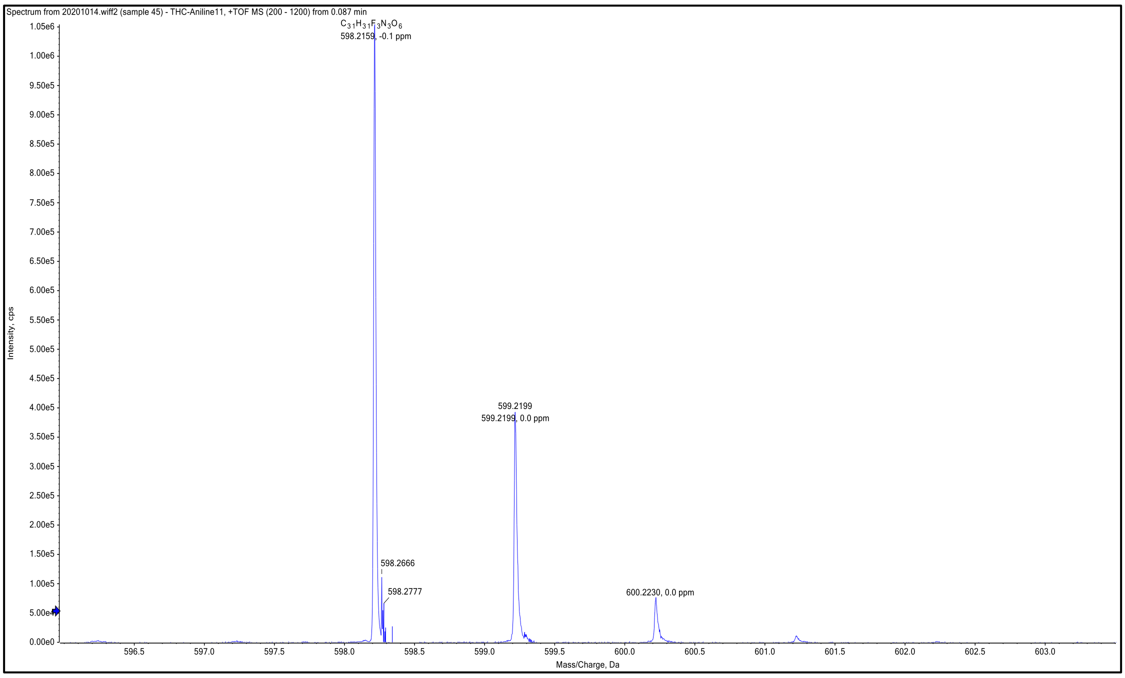

Figure S11. ^{1}H-NMR, ^{13}C-NMR, HR-ESI-MS spectrum of **4k**

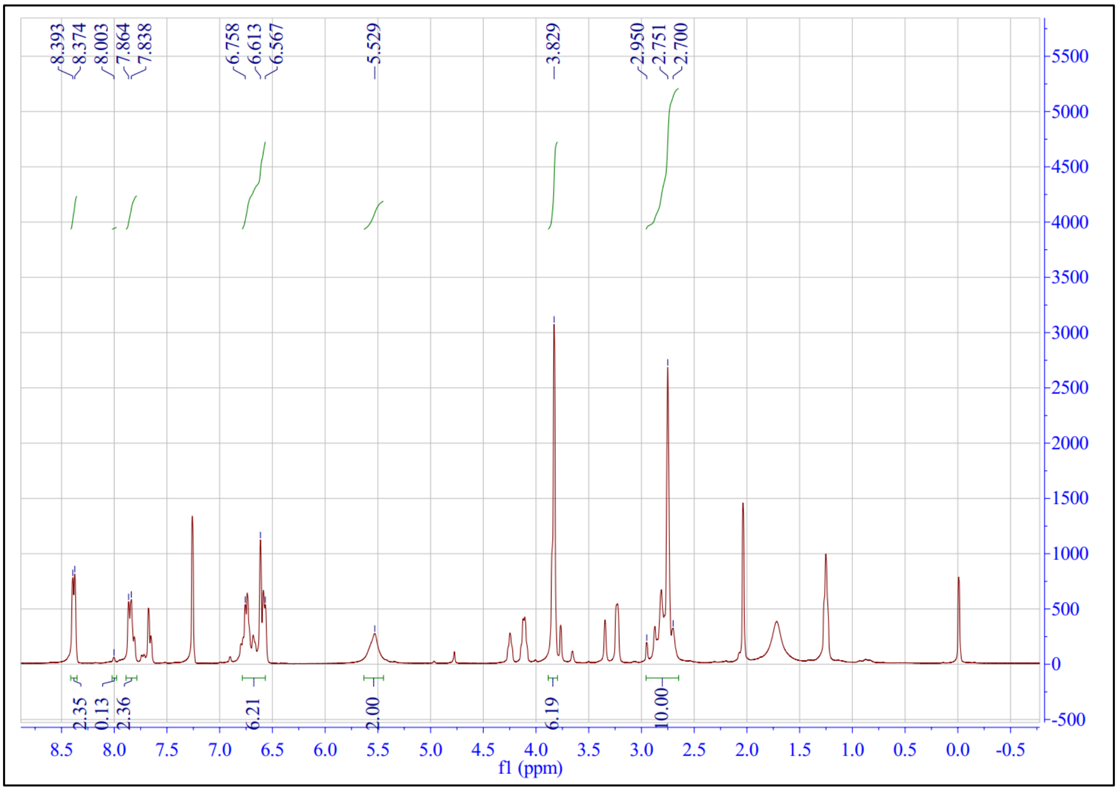

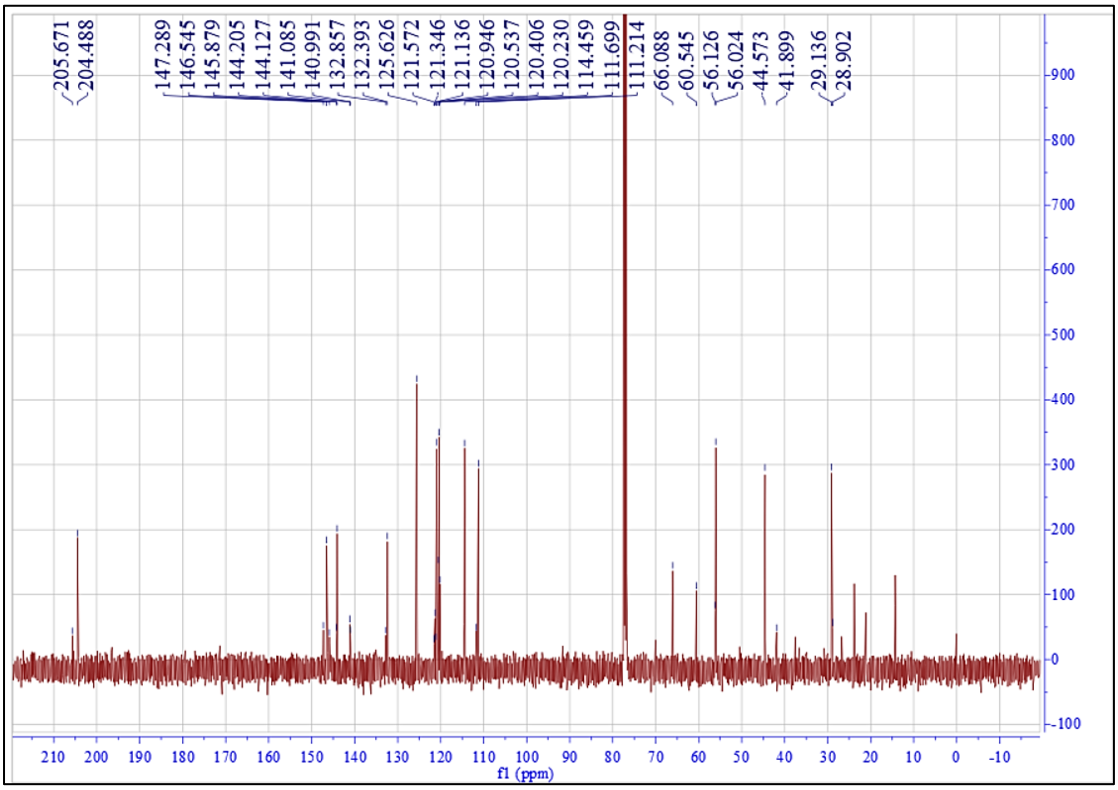

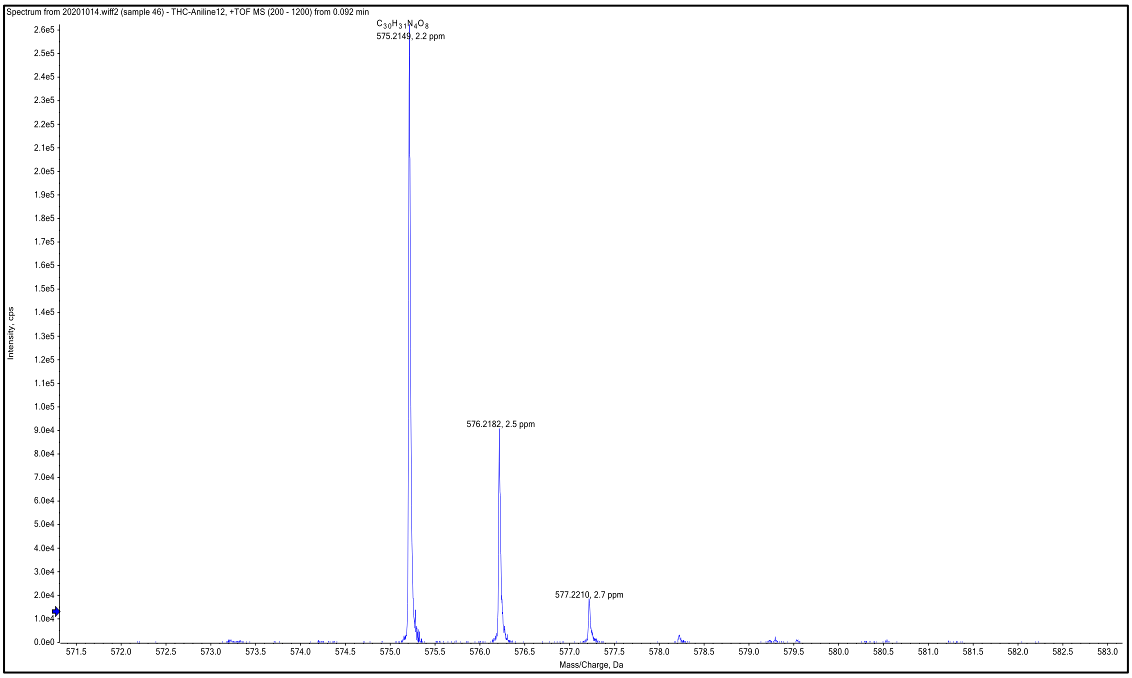

Figure S12. ^{1}H-NMR, ^{13}C-NMR, HR-ESI-MS spectrum of **4l**

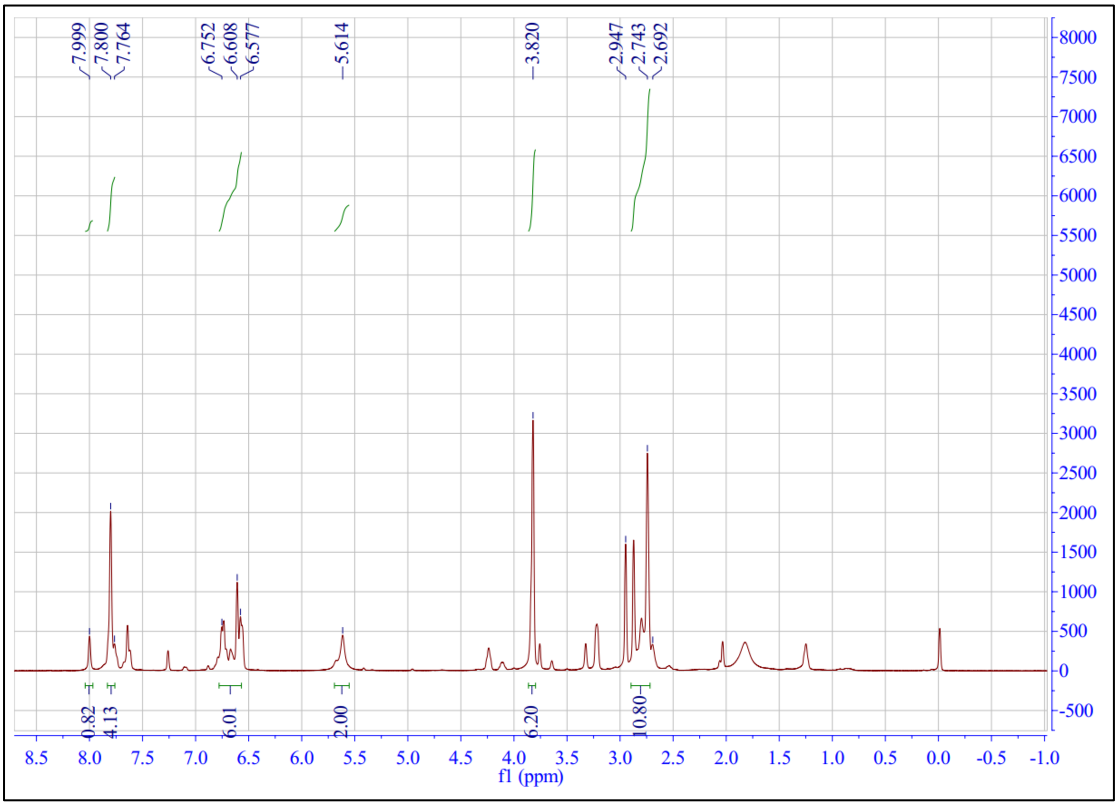
7.999
7.800
7.764
6.752
6.608
6.577
5.614
3.820
2.947
2.743
2.692
0.82
4.13
6.01
2.00
6.20
10.80
f1 (ppm)

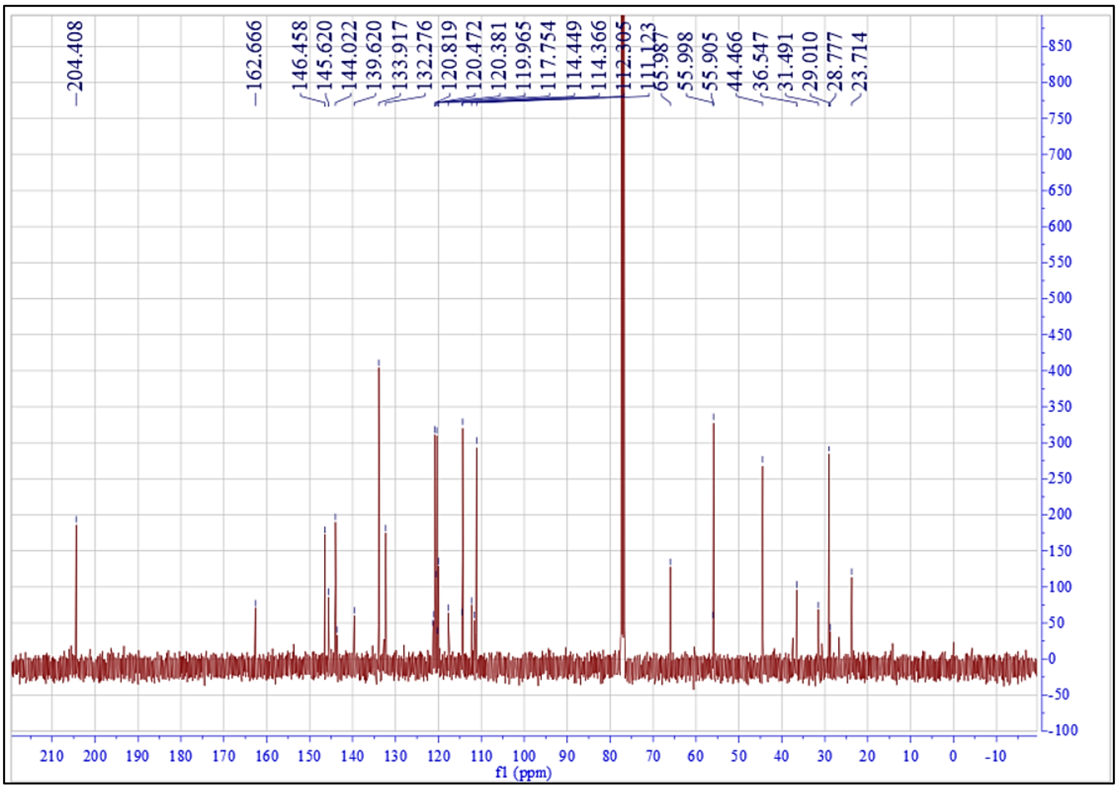
204.408
162.666
146.458
145.620
144.022
139.620
133.917
132.276
120.819
120.472
120.381
119.965
117.754
114.449
114.366
112.305
111.123
65.987
55.998
55.905
44.466
36.547
31.491
29.010
28.777
23.714
f1 (ppm)

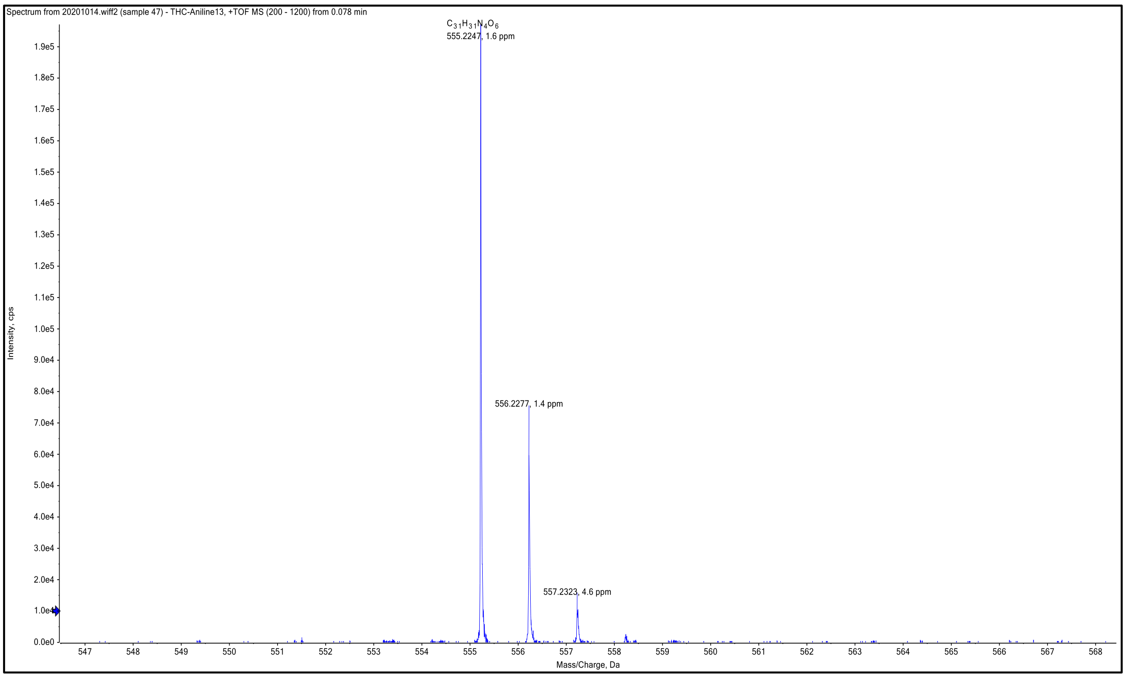

Figure S13. ^{1}H-NMR, ^{13}C-NMR, HR-ESI-MS spectrum of **4m**

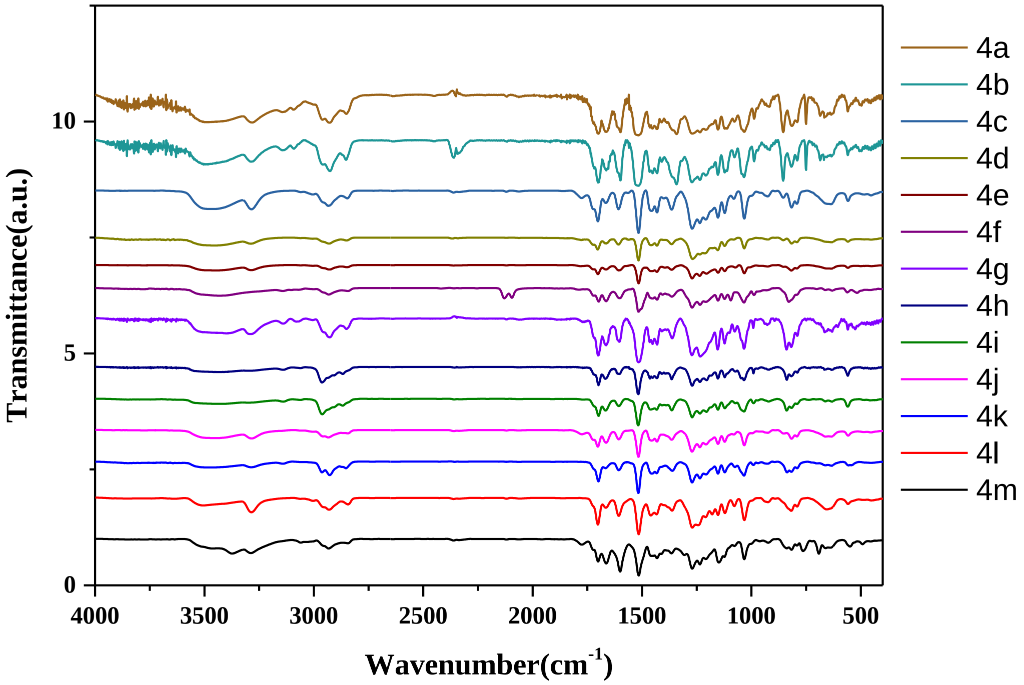

Figure S14. FTIR spectra of **4a~4m**
